# Beyond the limits of photoperception: constitutively active PHYTOCHROME B2 overexpression as a means of improving fruit nutritional quality in tomato

**DOI:** 10.1111/pbi.13362

**Published:** 2020-04-01

**Authors:** Frederico Rocha Rodrigues Alves, Bruno Silvestre Lira, Filipe Christian Pikart, Scarlet Santos Monteiro, Cláudia Maria Furlan, Eduardo Purgatto, Grazieli Benedetti Pascoal, Sónia Cristina da Silva Andrade, Diego Demarco, Magdalena Rossi, Luciano Freschi

**Affiliations:** ^1^ Departamento de Botânica Universidade de São Paulo São Paulo SP Brazil; ^2^ Departamento de Botânica Universidade Federal de Goiás Goiás GO Brazil; ^3^ Departamento de Alimentos e Nutrição Experimental Universidade de São Paulo São Paulo SP Brazil; ^4^ Curso de Graduação em Nutrição Universidade Federal de Uberlândia Minas Gerais MG Brazil; ^5^ Departamento de Genética e Biologia Evolutiva Universidade de São Paulo São Paulo SP Brazil

**Keywords:** photobiotechnology, *Solanum lycopersicum*, carotenoids, vitamin E, flavonoids, vitamin C, biofortification, antioxidants

## Abstract

Photoreceptor engineering has recently emerged as a means for improving agronomically beneficial traits in crop species. Despite the central role played by the red/far‐red photoreceptor phytochromes (PHYs) in controlling fruit physiology, the applicability of PHY engineering for increasing fleshy fruit nutritional content remains poorly exploited. In this study, we demonstrated that the fruit‐specific overexpression of a constitutively active GAF domain Tyr^252^‐to‐His PHYB2 mutant version (PHYB2^Y252H^) significantly enhances the accumulation of multiple health‐promoting antioxidants in tomato fruits, without negative collateral consequences on vegetative development. Compared with the native *PHYB2* overexpression, *PHYB2^Y252H^*‐overexpressing lines exhibited more extensive increments in transcript abundance of genes associated with fruit plastid development, chlorophyll biosynthesis and metabolic pathways responsible for the accumulation of antioxidant compounds. Accordingly, *PHYB2^Y252H^*‐overexpressing fruits developed more chloroplasts containing voluminous grana at the green stage and overaccumulated carotenoids, tocopherols, flavonoids and ascorbate in ripe fruits compared with both wild‐type and *PHYB2*‐overexpressing lines. The impacts of *PHYB2 *or *PHYB2^Y252H^* overexpression on fruit primary metabolism were limited to a slight promotion in lipid biosynthesis and reduction in sugar accumulation. Altogether, these findings indicate that mutation‐based adjustments in PHY properties represent a valuable photobiotechnological tool for tomato biofortification, highlighting the potential of photoreceptor engineering for improving quality traits in fleshy fruits.

## Introduction

Tomato (*Solanum lycopersicum* L.) is one of the most consumed vegetable crops in the world, both fresh or as processed juices and sauces (Bergougnoux, 2014). It is an important source of health‐promoting substances in the human diet, including carotenoids (e.g. lycopene and β‐carotene), flavonoids, ascorbic acid (i.e. vitamin C) and tocopherols (i.e. vitamin E) (Dorais *et al.*, 2008; Frusciante *et al.*, 2007). Accordingly, increasing attention has been devoted worldwide to improve tomato productivity and fruit quality traits both via traditional breeding or transgenic‐based approaches (Levin *et al.*, 2006; Liu *et al.*, 2004; Martin *et al.*, 2011).

Most attempts to improve fruit nutritional composition have focused on altering the expression of specific genes directly involved in the production of carotenoids, flavonoids and other health‐promoting substances (Gerszberg *et al.*, 2015). In contrast, the manipulation of key players of light perception and signalling transduction pathway has emerged as an alternative to promote more extensively tomato fruit biofortification by simultaneously altering the accumulation of multiple nutraceutical compounds (Azari *et al.*, 2010a; Davuluri *et al.*, 2005; Ganesan *et al.*, 2017; Gururani *et al.*, 2015).

Responsible for the red/far‐red light wavelength perception, phytochromes (PHYs) regulate a wide range of photomorphogenic responses throughout plant life cycle (Demotes‐Mainard *et al.*, 2016). Once photoactivated by red light exposure, PHYs allosterically change their conformation, triggering their translocation from cytosol to nucleus (Smith, 2000). In the nucleus, active PHYs interact with light signal transduction components, initiating highly complex and extensively interconnected signalling cascades that ultimately lead to the differential expression of photomorphogenesis‐related genes and proteasome‐dependent protein degradation events (Chen and Chory, 2011; Shin *et al.*, 2016). Upon far‐red light or dark exposure, active PHYs are converted back to the biologically inactive form (Seluzicki *et al.*, 2017). In tomato, five PHY‐encoding genes, namely *PHYA*, *PHYB1*, *PHYB2*, *PHYE* and *PHYF*, have been identified (Alba *et al.*, 2000a). *PHYB2* is the most expressed in tomato fruits (Bianchetti *et al.*, 2017; Hauser *et al.*, 1997; Pratt *et al.*, 1995) and a major regulator of fruit chloroplast maturation and carotenoid accumulation (Bianchetti *et al.*, 2018; Gupta *et al.*, 2014).

Besides the mRNA levels, PHY protein activity can also be manipulated. In *Arabidopsis thaliana* and *Avena sativa*, specific amino acid changes in the photosensor module domains result in a light‐independent constitutive biological activity (Jeong *et al.*, 2016; Su and Lagarias, 2007). This is the case of the mutation Tyr^276^‐to‐His in the conserved GAF domain of *Arabidopsis* PHYB that renders constitutive PHY‐dependent photomorphogenic responses, even in the darkness (Hu *et al.*, 2009; Oka *et al.*, 2011; Su and Lagarias, 2007).

Here, we investigated whether the overexpression of the constitutively active GAF domain Tyr‐to‐His PHYB2 mutant version (PHYB2^Y252H^) is suitable to improve PHY‐dependent developmental and metabolic responses associated with tomato fruit nutritional composition, such as plastid development (Bianchetti *et al.*, 2017, 2018), carotenogenesis (Alba *et al.*, 2000b; Bianchetti *et al.*, 2018; Gupta *et al.*, 2014) and tocopherol biosynthesis (Gramegna *et al.*, 2018). To circumvent adverse collateral effects on vegetative growth (e.g. reduced plant height, apical dominance and leaf size) normally caused by whole‐plant *PHY* overexpression (Garg *et al.*, 2006; Husaineid *et al.*, 2007; Thiele *et al.*, 1999), a fruit‐specific overexpression strategy was employed. Our data indicated that the expression of the constitutively active PHYB2^Y252H^ promotes the biosynthesis and accumulation of carotenoids, flavonoids and vitamins C and E in tomato fruits to significantly higher levels than those detected when the native PHYB2 was overexpressed. Fruit plastid biogenesis and differentiation were particularly promoted by *PHYB2^Y252H^* overexpression, accompanied by an overall up‐regulation of genes encoding chloroplast components and photosynthesis‐related proteins. Fruit primary metabolism was only marginally affected by the fruit‐specific *PHYB2* or *PHYB2^Y252H^* overexpression, which further highlights the potential application of this genetic manipulation to enhance tomato fruit nutritional quality.

## Results

### Fruit‐specific *PHYB2* and *PHYB2^Y252H^* overexpression in tomato plants

**Figure 1:**
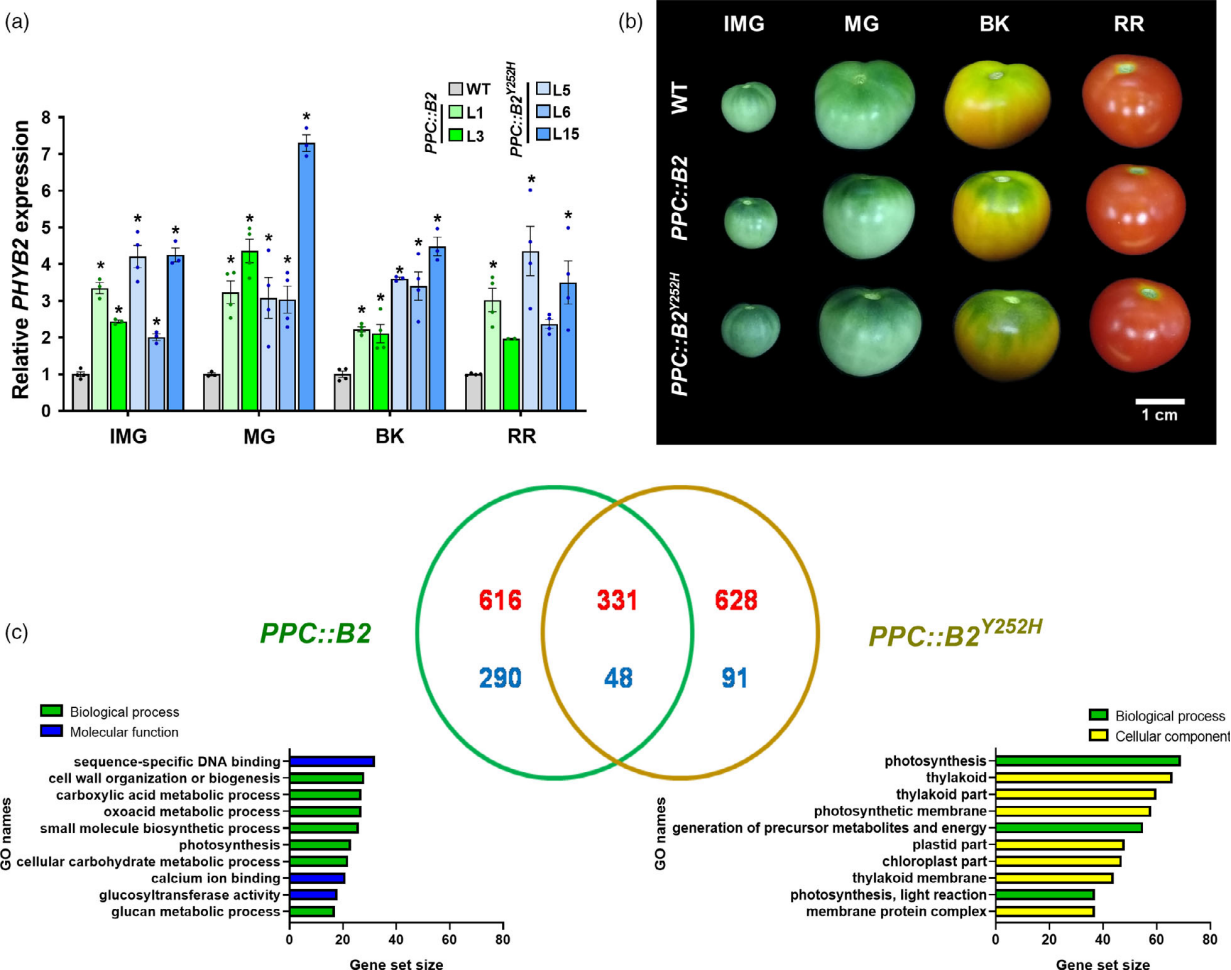
*PHYB2* and *PHYB2^Y252H^* overexpression modify fruit pigmentation and global transcriptomic profile. (a) *PHYB2* or *PHYB2^Y252H^* mRNA levels throughout fruit development of the tomato (Micro‐Tom cultivar) transgenic lines. Transcript abundance was normalized against *PHYB2* mRNA levels detected in wild‐type (WT) at each stage. Data are mean ± SE, and dots represent individual values. Statistical differences within each stage are given by asterisks (Dunnett’s test with WT as control group, α = 0.05). (b) Visual phenotype of transgenic fruits in different development stages. (c) Venn's diagram analysis and Gene Set Enrichment Analysis (GSEA) of exclusive differentially expressed genes (DEGs) of *PPC2::PHYB2* (*PPC::B2*) and *PPC2::PHYB2^Y252H^* (*PPC::B2^Y252H^*) BK fruits compared with WT counterparts. Up‐ and down‐regulated genes are indicated in red and blue colours, respectively. Numbers represent overlapping changes among DEGs (FDR < 0.05). CPM values and GO terms are detailed in Tables S2 and S3, respectively. IMG, immature green; MG, mature green; BK, breaker; RR, red ripe.

To identify the conserved tyrosine residue to generate hyperactive GAF domain Tyr‐to‐His PHYB2 mutants, the coding sequence of *Solanum lycopersicum* PHYB2 was aligned and compared with *Arabidopsis thaliana* PHYB. The conserved Y^276^ residue of the *Arabidopsis* PHYB GAF domain corresponds to Y^252^ in tomato PHYB2 (Figure S1). Next, transgenic lines of tomato (Micro‐Tom cultivar) expressing the native *PHYB2* gene or its mutant hyperactive form *PHYB2^Y252H^* under the control of fruit‐specific *PHOSPHOENOLPYRUVATE CARBOXYLASE 2* (*PPC2*) promoter (Fernandez *et al.*, 2009) were generated. Overexpression of the transgenes was confirmed by RT‐qPCR in two *PPC2::PHYB2* (*PPC::B2*) lines (namely L1 and L3) and three *PPC2::PHYB2^Y252H^* (*PPC::B2^Y252H^*) lines (namely L5, L6 and L15) (Figure 1a). *PHYB2* or *PHYB2^Y252H^* transcript levels were between twofold and sevenfold higher in the transgenic lines compared with *PHYB2* mRNA levels detected in WT fruits throughout initial fruit development (immature green, IMG; mature green, MG) and ripening stages (breaker, BK; red ripe, RR) (Figure 1a). Transcript abundance of other *PHY* genes remained virtually indistinguishable between the WT and transgenic lines throughout fruit development and ripening (Table S1).

The fruit‐specific overexpression of either *PHYB2* or *PHYB2^Y252H^* resulted in no apparent phenotypical alterations on plant vegetative growth (Figure S2). In contrast, green fruits (IMG and MG) from the *PPC::B2^Y252H^* lines exhibited significantly darker green coloration at the fruit pedicellar (shoulder) region than WT counterparts (Figure 1b). At the BK stage, the green shoulder phenotype persisted in both *PPC::B2* and *PPC::B2^Y252H^* fruits, whereas it was significantly attenuated in WT fruits. The green shoulder phenotype was subsequently lost in all genotypes at the RR stage (Figure 1b).

RNA‐seq transcriptomic profiling performed in BK fruits of representative *PPC::B2* (L1) and *PPC::B2^Y252H^* (L6) lines revealed that up to 5.4% of the 23 685 transcriptionally active genes at this developmental stage was affected either by the *PHYB2* or *PHYB2^Y252H^* overexpression. Compared with the WT, 906 and 719 genes were differentially expressed exclusively in *PPC::B2* and *PPC::B2^Y252H^* fruits, respectively. Only 379 differentially expressed genes (DEGs) were common in both transgenics when compared to the WT, with a predominance of up‐regulated (331) over down‐regulated (48) genes (Figure 1c, Table S2). RNA‐seq results were further validated by RT‐qPCR analysis, demonstrating consistency (*r* ^2^ correlation above 0.9) between both methods (Figure S3).

According to the Gene Set Enrichment Analysis (GSEA), the exclusive *PPC::B2* DEGs were predominantly associated with global cell functioning and diverse metabolic processes, grouping around GO terms such as sequence‐specific DNA binding, cell wall organization or biogenesis, oxoacid metabolic process and small molecule biosynthetic process (Figure 1c, Table S3). In contrast, the majority of DEGs exclusively detected in *PPC::B2^Y252H^* fruits were associated with chloroplast components and related processes, including GO terms as photosynthesis, thylakoid, photosynthetic membrane and generation of precursor metabolites and energy (Figure 1c, Table S3).

### Either *PHYB2* or *PHYB2^Y252H^* overexpression promotes fruit plastid biogenesis but only *PHYB2^Y252H^* enhances chlorophyll accumulation

**Figure 2:**
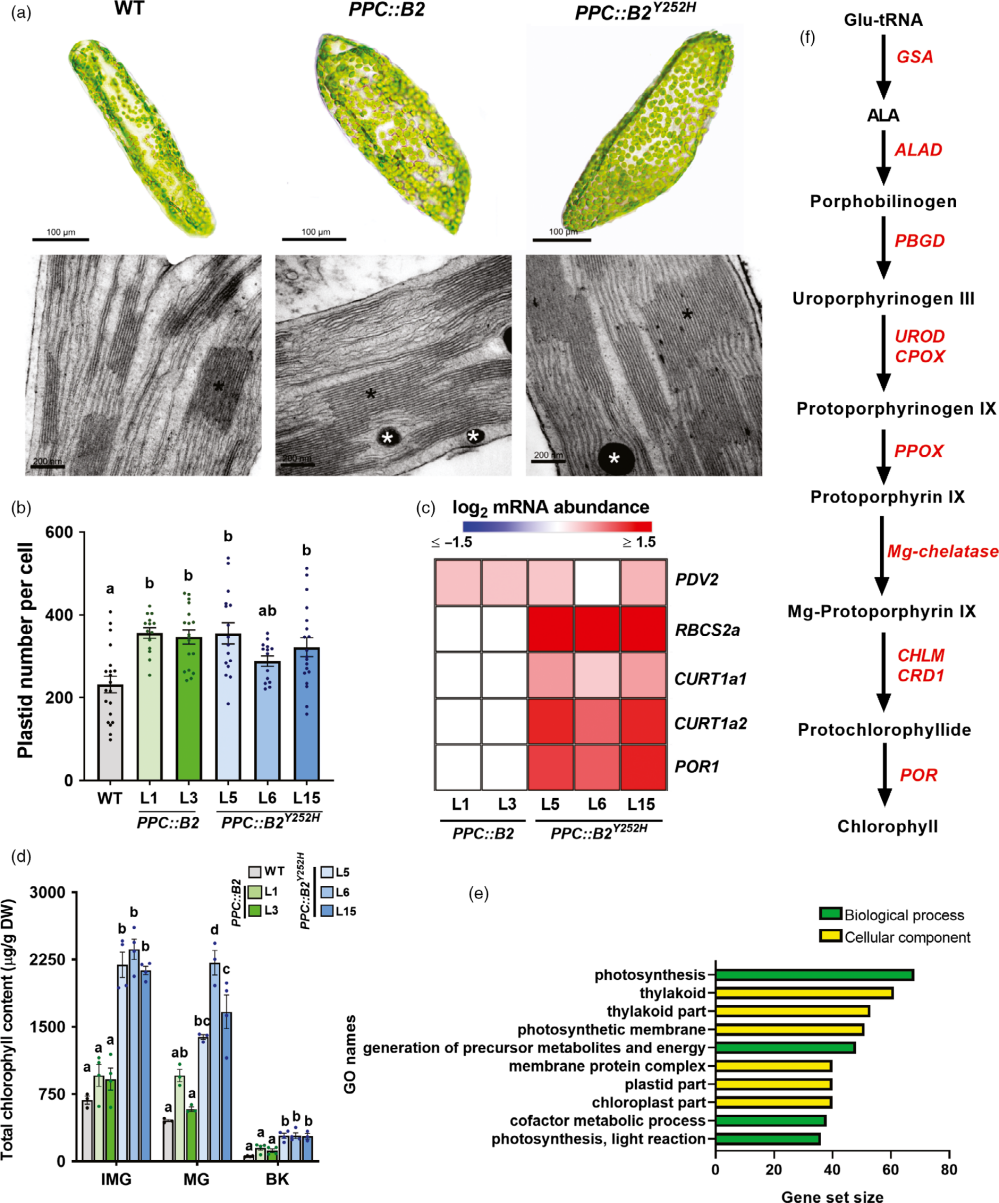
Fruit‐specific *PHYB2* and *PHYB2^Y252H^* overexpression promote chloroplast biogenesis and differentiation and chlorophyll biosynthesis. (a) Representative optical microscopy (upper panels) and transmission electron microscopy images (bottom panels) of plastids at the pedicel region of immature green (IMG) fruits of wild‐type (WT), *PPC2::PHYB2* (*PPC::B2*) and *PPC2::PHYB2^Y252H^* (*PPC::B2^Y252H^*) plants of Micro‐Tom cultivar. Black asterisks indicate stacked thylakoids and white asterisks indicate plastoglobules. (b) Plastid number per pericarp cell of WT, *PPC::B2* and *PPC::B2^Y252H^* fruits at mature green (MG) stage. (c) Heatmap representation of the statistically significant differences in mRNA abundance of plastid‐related genes normalized against WT transcript levels at IMG stage (Dunnett's test, α = 0.05) via RT‐qPCR. Gene abbreviations and relative transcript values are detailed in Table S4. (d) Total chlorophyll content of WT, *PPC::B2* and *PPC::B2^Y252H^* fruits. (e) Gene Set Enrichment Analysis (GSEA) of exclusive differentially expressed genes (DEGs) of *PPC::B2^Y252H^* MG fruits compared with WT counterparts. GO terms are detailed in Table S3. (f) Simplified chlorophyll biosynthetic pathway. Intermediate reactions are omitted. Up‐regulated chlorophyll biosynthesis‐related genes in *PPC::B2^Y252H^* MG fruits according to RNA‐seq analysis are highlighted in red. Gene abbreviations, as well as logFC and FDR values, are detailed in Table S6. In (b) and (d), data are mean ± SE, dots represent individual values, and statistical differences within each stage are given by different letters (Tukey's Test, α = 0.05). BK, breaker; ALA, 5‐aminolevulinic acid.

The greener fruit phenotype and up‐regulation of plastid‐related genes in the transgenic lines prompted us to investigate the impacts of either *PHYB2* or *PHYB2^Y252H^* overexpression on fruit chloroplast biogenesis and development. Microscopy analysis revealed increments of up to 50% in plastid abundance in pericarp cells of *PPC::B2* and *PPC::B2^Y252H^* green fruits compared with WT counterparts (Figure 2a,b). The increased number of plastids detected in *PPC::B2* and *PPC::B2^Y252H^* fruits was associated with up‐regulation of *PLASTID DIVISION 2 *(*PDV2*) (Figure 2c), which encodes a vital component of the plastid division machinery (Okazaki *et al.*, 2009).

Increased thylakoid stacking with more voluminous grana was observed in plastids of *PPC::B2* green fruits, a phenotype further intensified in *PPC::B2^Y252H^* fruits (Figure 2a, Figure S4). In agreement, transcript abundances of several photosynthesis‐ and plastid‐related genes, including those encoding Rubisco subunits (i.e. *RBCS2a*), were up‐regulated in IMG fruits of *PPC::B2^Y252H^* lines, but not in *PPC::B2* fruits (Figure 2c). Similarly, two tomato homologs of *Arabidopsis* mediator of thylakoid membrane bending at the grana margins *CURVATURE THYLAKOID 1a* (*CURT1a*, Armbruster *et al.*, 2013), namely *CURT1a1* (Solyc01g095430) and *CURT1a2* (Solyc10g011770) (Figure S5), were also exclusively up‐regulated in *PPC::B2^Y252H^* lMG fruits (Figure 2c). In contrast, mRNA levels encoding transcription factors previously associated with altered plastid maturation and maintenance in green tomato fruits, such as *GOLDEN 2‐LIKE 2* (*GLK2*), *AUXIN RESPONSE FACTOR 4* (*ARF4*), *ARABIDOPSIS PSEUDORESPONSE REGULATOR 2‐LIKE* (*APRR2Like*) and *BEL‐1 LIKE HOMEODOMAIN 11* (*BEL11*) were virtually indistinguishable between the wild‐type and transgenic lines (Table S4).

Total chlorophyll content was about threefold higher in *PPC::B2^Y252H^* green fruits than in the WT (Figure 2d, Table S5), which was associated with increments of approximately twofold in transcript levels of *PROTOCHLOROPHYLLIDE OXIDOREDUCTASE 1* (*POR1*) (Figure 2c), which encodes an essential light‐triggered chlorophyll biosynthesis‐related enzyme (Heyes and Hunter, 2005). Moreover, transcriptome analysis carried out in MG fruits revealed enrichment of transcripts associated with photosynthesis, thylakoid and plastid membranes in *PPC::B2^Y252H^* compared with WT fruits (Figure 2e). Most chlorophyll biosynthetic genes were significantly up‐regulated in *PPC::B2^Y252H^* MG fruits compared with the WT (Figure 2f, Table S6), which agrees with the higher chlorophyll content detected in the transgenic fruits. In contrast, *PPC::B2* green fruits showed no significant changes in total chlorophyll levels nor in *POR1* mRNA levels compared with the WT (Figure 2c,d, Tables S4 and S5).

### *PHYB2^Y252H^* overexpression promotes fruit isoprenoid metabolism

Besides photosynthesis, chloroplasts are essential organelles for the synthesis and storage of secondary metabolites (Armbruster *et al.*, 2011). Therefore, increments in fruit plastid abundance and size have consistently been associated with increased tomato fruit nutritional composition (Bianchetti *et al.*, 2017; Bino *et al.*, 2005; Cocaliadis *et al.*, 2014; Cruz *et al.*, 2018; Enfissi *et al.*, 2010; Galpaz *et al.*, 2008; Kolotilin *et al.*, 2007; Lupi *et al.*, 2019; Nguyen *et al.*, 2014). Besides, light signalling can also directly influence the expression of genes involved in the production of isoprenoids (e.g. carotenoids, tocopherols) and other health‐promoting substances accumulated in tomato fruits (Alba *et al.*, 2000b; Gramegna *et al.*, 2018; Ksas *et al.*, 2015; Llorente *et al.*, 2016).

**Figure 3:**
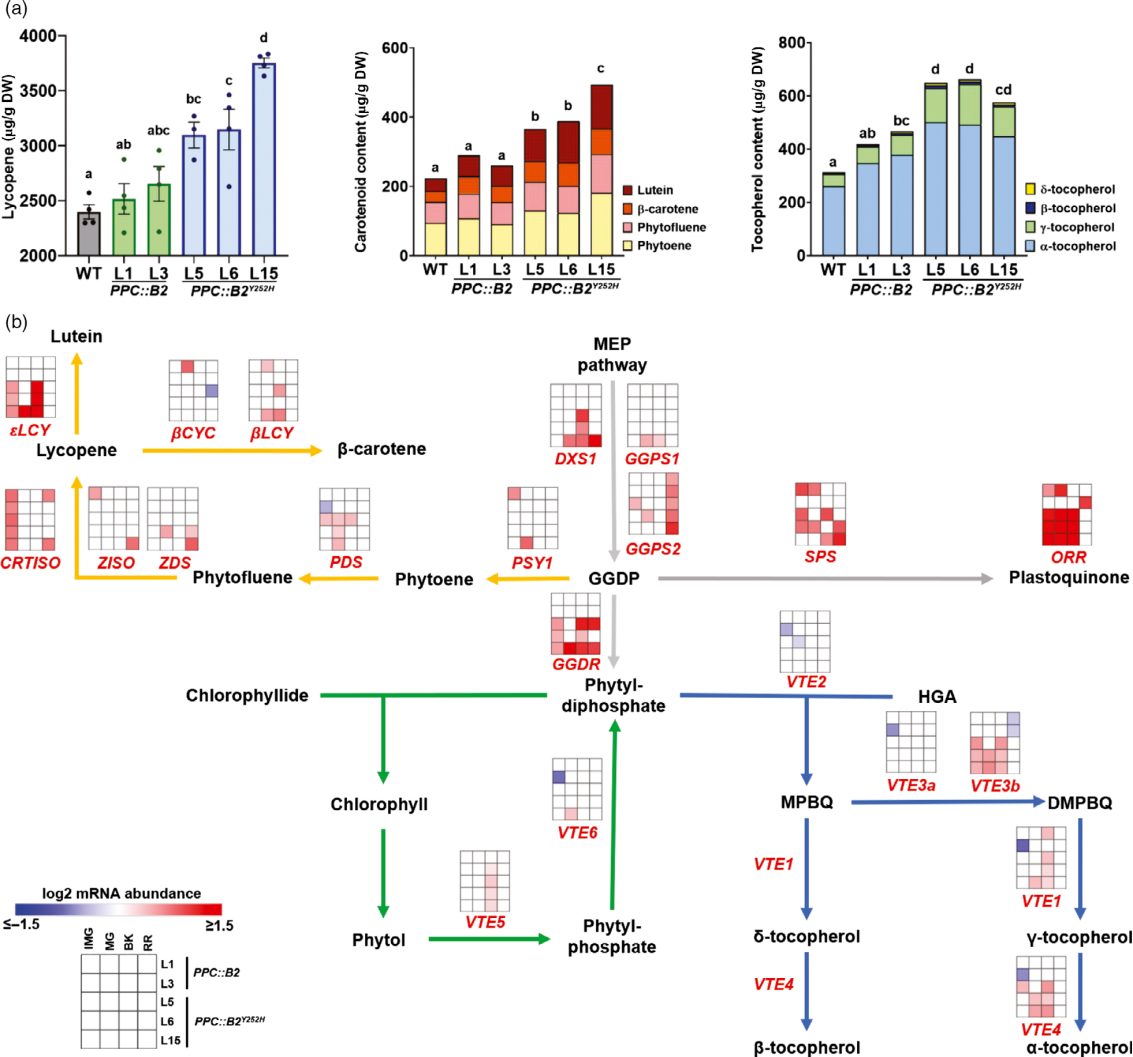
Fruit‐specific *PHYB2^Y252H^* overexpression promotes isoprenoid metabolism. (a) Carotenoid (lycopene, phytoene, phytofluene, lutein and β‐carotene) and tocopherol (α, β, δ and γ forms) content in wild‐type (WT), *PPC2::PHYB2* (*PPC::B2*) and *PPC2::PHYB2^Y252H^* (*PPC::B2^Y252H^*) red ripe (RR) fruits of Micro‐Tom cultivar. Data are mean ± SE, and dots represent individual values. Statistical differences are given by different letters (Tukey's test, α = 0.05). (b) Schematic representation of isoprenoid metabolism interconnecting carotenoid (orange), tocopherol (blue) and chlorophyll (green) pathways. Intermediate reactions are omitted. Heatmap representation of the statistically significant differences in mRNA abundance of isoprenoid‐related genes normalized against WT transcript levels at each stage (Dunnett's test, α = 0.05). Gene abbreviations and relative transcript values are detailed in Table S7. IMG, immature green; MG, mature green; BK, breaker; MEP, methylerythritol phosphate; GGDP, geranylgeranyl diphosphate; HGA, homogentisic acid; MBPQ, 2‐methyl‐6‐phytyl‐1,4‐benzoquinone; DMBPQ, 2,3‐dimethyl‐6‐phytyl‐1,4‐benzoquinone.

Carotenoid and tocopherol profiling revealed that *PHYB2* overexpression promoted exclusively β‐carotene and lutein accumulation in RR fruits (Figures 3a and S6, Table S5). In contrast, *PPC::B2^Y252H^* RR fruits exhibited significant increments in virtually all individual carotenoids analysed, with increments of about 50% in phytoene, phytofluene and lycopene levels and between 100% and 200% in β‐carotene and lutein levels, respectively. Total tocopherol content in red fruits exhibited a similar trend, with increments of up to twofold in *PPC::B2^Y252H^* lines (Figures 3a and S6, Table S5). The levels of α‐tocopherol, the dominant tocopherol form in tomato fruits, as well as β‐carotene and lutein contents were significantly higher in *PHYB2^Y252H^*‐overexpressing lines than in the WT at all sampled stages (Table S5). This indicates that the PHYB2^Y252H^‐triggered intensification of fruit isoprenoid accumulation was not restricted to the ripening stages.

Transcript levels of many isoprenoid biosynthetic genes were up‐regulated in response to *PHYB2* and *PHYB2^Y252H^* overexpression (Figure 3b). Genes encoding rate‐limiting enzymes responsible for the synthesis of isoprenoid precursor geranylgeranyl diphosphate (GGDP), such as *1‐DEOXY‐D‐XYLULOSE‐5‐PHOSPHATE SYNTHASE 1* (*DXS1*) and *GGDP SYNTHASE 2* (*GGPS2*), were up‐regulated in *PPC::B2^Y252H^* and in all transgenic lines, respectively. In contrast, except *LYCOPENE Ɛ‐CYCLASE* (*εLCY*), other core carotenoid biosynthesis genes were not consistently up‐regulated in the transgenic fruits during ripening. Likewise, mRNA levels of transcription factors associated with the ripening‐related regulation of carotenoid biosynthesis, such as *RIPENING INHIBITOR* (*RIN*), *NON‐RIPENING* (*NOR*), *COLORLESS NON‐RIPENING* (*CNR*), *APETALA2a* (*AP2a*) and *FRUITFULL1* (*FUL1*) (Liu *et al.*, 2015), were also unaltered in the transgenic lines during fruit ripening (Table S7).

The activities of the carotenoid desaturase enzymes PHYTOENE DESATURASE (PDS) and ζ‐CAROTENE DESATURASE (ZDS) are especially sensitive to the cellular redox state, requiring plastoquinones (PQs) as co‐factors to accept electrons produced during the desaturation steps of GGDP to lycopene (Fanciullino *et al.*, 2014; Norris *et al.*, 1995). The transcript levels of the PQ biosynthetic enzyme *SOLANESYL DIPHOSPHATE SYNTHASE* (*SPS*) were up‐regulated in *PPC::B2^Y252H^* lines, likely boosting PQ biosynthesis in the transgenic fruits. Moreover, *ORANGE RIPENING* (*ORR*), a gene encoding a NADH dehydrogenase (NdH) complex subunit responsible for PQ reduction (Endo *et al.*, 2008), was also up‐regulated in *PPC::B2^Y252H^* lines (Figure 3b).

*GERANYLGERANYL DIPHOSPHATE REDUCTASE* (*GGDR*), which encodes the enzyme responsible for the *de novo* synthesis of tocopherol precursor phytyl diphosphate (PDP), was markedly up‐regulated exclusively in *PPC::B2^Y252H^* fruits at all sampling stages, suggesting increased conversion of GGDP to PDP in these transgenic lines. During green stages of fruit development, GGDR up‐regulation presumably promoted PDP production, feeding the enhanced chlorophyll biosynthetic pathway mentioned above (Figure 3b). From the onset of ripening onwards, chlorophyll biosynthesis ceases and the precursor GGDP are channelled towards carotenoid production by the transcriptional inhibition of *GGDR* in WT (Table S7, Quadrana *et al.*, 2013), whereas chlorophyll degradation releases phytol, which is, in part, incorporated into tocopherol (Zhang *et al.*, 2014), leading to its increment during ripening (Table S5). Interestingly, at the BK stage in *PPC::B2^Y252H^* lines, *PHYTOL KINASE* (*VTE5*), which is responsible for the first step of phytol phosphorylation (Valentin *et al.*, 2006), was up‐regulated compared with WT counterparts (Figure 3b), probably enhancing the production of PDP and contributing to the increase in total tocopherol content observed all along fruit development (Figure 3b). Accordingly, tocopherol biosynthetic genes, particularly *2,3‐DIMETHYL‐5‐PHYTYLQUINOL METHYLTRANSFERASE b* (*VTE3b*), *TOCOPHEROL CYCLASE* (*VTE1*) and *TOCOPHEROL C‐METHYL TRANSFERASE* (*VTE4*), were also predominantly up‐regulated in *PPC::B2^Y252H^* lines from the IMG to the BK stage (Figure 3b).

### Both flavonoid and ascorbate metabolisms are up‐regulated in *PHYB2^Y252H^* ripe fruits

**Figure 4:**
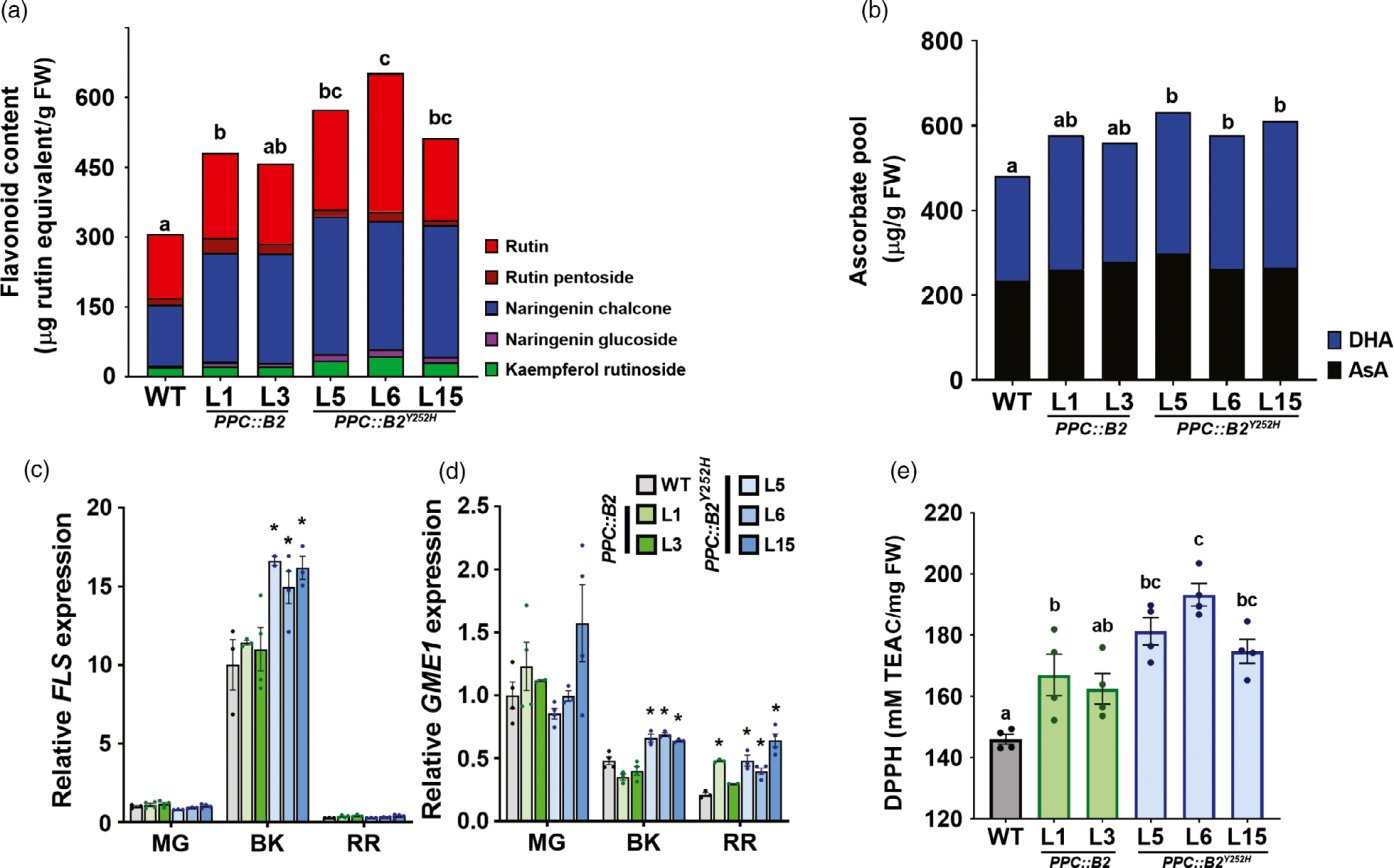
Flavonoid and ascorbate levels are increased in *PHYB2^Y252H^*‐overexpressing fruits. (a) Flavonoid content in wild‐type (WT), *PPC2::PHYB2* (*PPC::B2*) and *PPC2::PHYB2^Y252H^* (*PPC::B2^Y252H^*) red ripe (RR) fruits of Micro‐Tom cultivar. (b) Total ascorbate pool in RR fruits. (c,d) *FLS* and *GME1* mRNA levels throughout fruit development. Transcript abundance was normalized against wild‐type (WT) samples at the mature green (MG) stage. (e) Total antioxidant activity of polar extracts expressed as Trolox Equivalent Antioxidant Capacity (TEAC) in RR fruits. Data are mean ± SE, and dots represent individual values. Metabolites, gene abbreviations and relative transcript values are detailed in Tables S8 and S9. Statistical differences within each stage are given by asterisks (Dunnett's test with WT as control group, α = 0.05) or different letters (Tukey's test, α = 0.05). DHA, dehydroascorbate; AsA, ascorbic acid; BK, breaker.

In our study, the fruit‐specific *PHYB2^Y252H^* overexpression significantly promoted both flavonoid and ascorbate contents in RR fruits, whereas the levels of these antioxidants were slightly, but not significantly, increased in *PPC::B2* fruits. Increments between twofold and threefold in rutin, kaempferol rutinoside, naringenin chalcone and naringenin glucoside contents were registered in *PPC::B2^Y252H^* RR fruits compared with WT counterparts (Figures 4a and S7, Table S8). Both ascorbate and dehydroascorbate levels were overaccumulated in *PPC::B2^Y252H^* fruits (Figure S7), resulting in increments of approximately 20% in the total pool of this antioxidant (Figure 4b).

On the other hand, the *PPC::B2^Y252H^*‐induced increments in rutin, which is a major flavonoid accumulated in tomato fruits (Slimestad and Verheul, 2009), as well as kaempferol rutinoside, were associated with significantly higher transcript abundance of *FLAVONOL SYNTHASE* (*FLS*) in the transgenic fruits at the BK stage (Figure 4c). The higher ascorbate content was associated with increased transcript levels of *GDP‐D‐MANNOSE‐3,5‐EPIMERASE 1* (*GME1*) (Figure 4d), which encodes an enzyme responsible for an initial step in the ascorbate biosynthetic pathway (Wolucka and Van Montagu, 2007). RNA‐seq data revealed that *GME1* was the only core ascorbate biosynthetic gene modulated by *PHYB2^Y252H^* overexpression (Figure S8), with up to twofold increments in mRNA levels at the BK and RR stages as confirmed by qPCR analysis (Figure 4d).

Consistent with the differential effect of *PHYB2* and *PHYB2^Y252H^* overexpression on isoprenoid flavonoids and ascorbate metabolism in RR fruits, only *PPC::B2^Y252H^* lines consistently exhibited higher total antioxidant activity, as estimated by the DPPH assay, reaching increments of up to 25% compared with WT counterparts (Figure 4e, Table S8).

### Lipid and sugar metabolisms are the main primary metabolic pathways affected in the transgenic fruits

**Figure 5:**
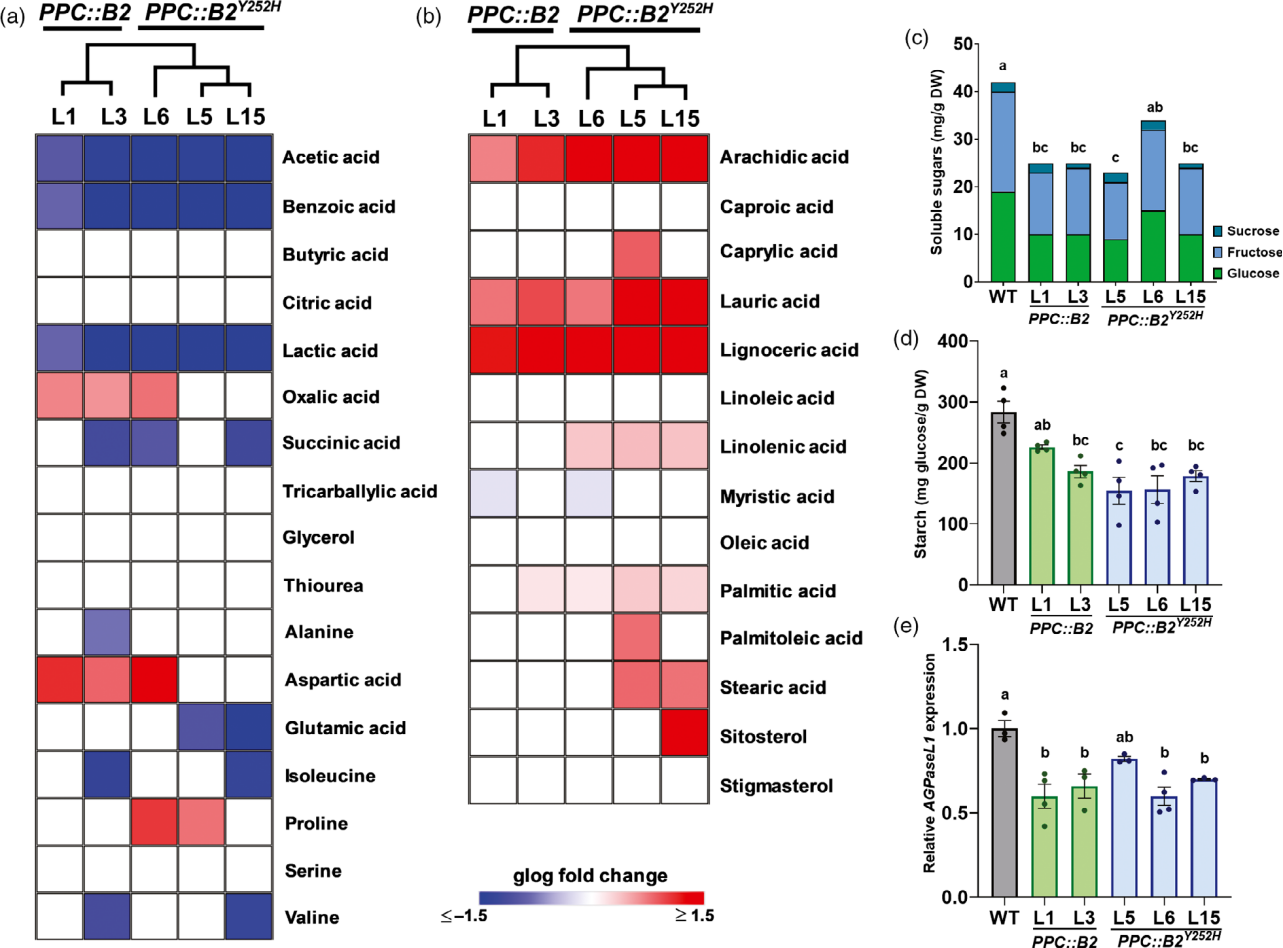
Fruit‐specific *PHYB2* and *PHYB2^Y252H^* overexpression impacts on primary metabolism are limited to changes in lipid and sugar contents. (a,b) Heatmap representation of the relative abundance of polar (a) and apolar (b) compounds in *PPC2::PHYB2* (*PPC::B2*) and *PPC2::PHYB2^Y252H^* (*PPC::B2^Y252H^*) red ripe (RR) transgenic fruits of Micro‐Tom cultivar. Only statistically different results are displayed in generalized logarithm (glog) normalized against the wild‐type (WT) (Dunnett's test, α = 0.05). Relative metabolite abundances are detailed in Table S10. Dendrograms indicate hierarchical clustering relationships between lines. (c) Soluble sugars (sucrose, glucose and fructose) content in RR fruits. (d) Starch content in immature green (IMG) fruits. (e) *AGPaseL1* mRNA levels in IMG fruits. In (d,e), data are mean ± SE and dots represent individual values. Metabolites, gene abbreviations and relative transcript values are detailed in Tables S11 and S13. Statistical differences are given by different letters (Tukey's test, α = 0.05).

Mass spectrometry (MS)‐based metabolite profiling and high‐performance liquid chromatography (HPLC)‐based soluble carbohydrate (sucrose, glucose, fructose) and organic acid (citric acid) analysis were used to investigate the impacts of *PHYB2* or *PHYB2^Y252H^* overexpression on primary metabolism of ripe fruits. About 25% of the metabolites identified via GC–MS profiling was consistently altered in the transgenic lines compared with WT counterparts (Table S10). Hierarchical clustering analysis supported the differences between the clades composed by the *PPC::B2* and *PPC::B2^Y252H^* lines based on polar and apolar metabolite profiles, evidencing that fruit metabolome was differentially affected by *PHYB2* or *PHYB2^Y252H^* overexpression (Figure 5a‐b).

Minor organic acids such as acetic, benzoic, lactic and succinic acids were under‐accumulated in all transgenic lines, whereas oxalic acid was overaccumulated in *PPC::B2* lines. Citric acid, which is the most abundant organic acid in tomato (Morgan *et al.*, 2013), was not significantly altered in transgenic fruits throughout fruit development and ripening (Table S11). Except for aspartic acid, aminoacids were not consistently altered in the transgenic fruits compared with WT counterparts (Figure 5a).

Saturated fatty acids such as arachidic, lauric and lignoceric acids were significantly more abundant in all transgenic lines, whereas the levels of the polyunsaturated ω‐6 linolenic acid were consistently increased only in *PPC::B2^Y252H^* fruits (Figure 5b). RNA‐seq data analysis revealed that some genes encoding important lipid metabolism‐related enzymes are up‐regulated, particularly in *PPC::B2^Y252H^* lines (Table S2). For instance, *GLYCEROL‐3‐PHOSPHATE ACYLTRANSFERASE* (Solyc08g076470), which encodes the enzyme catalysing an initial reaction in the biosynthesis of phospholipids (Chen *et al.*, 2011), and some *FATTY ACID DESATURASES* (FADs, Solyc06g051400 and Solyc07g005510) encoding genes, associated with the accumulation of polyunsaturated fatty acids (Dar *et al.*, 2017), were up‐regulated in the *PPC::B2^Y252H^* (Table S2).

Glucose, fructose and sucrose contents were indistinguishable in WT and transgenic green fruits (Table S11). However, glucose and fructose levels were predominantly lower in the RR transgenic fruits than in the WT counterparts, whereas sucrose remained at similar levels in all genotypes (Figure 5c and S9, Table S11). Accordingly, total soluble solids content (ºBrix) values in transgenic ripe fruits were approximately 5% lower than in the WT counterparts (Figure S9). The amount of soluble sugars in ripe tomato fruits is determined by several factors, including the plant photosynthetic capacity, dynamics of starch biosynthesis and breakdown, and sucrose import from vegetative tissues and conversion into hexoses (Patrick *et al.*, 2013). To investigate the possible causes of the reduced levels of soluble sugars in the transgenic lines, we first estimated the plant photosynthetic capacity by measuring gas exchange, fluorescence parameters, chlorophyll content and starch levels in source leaves of all genotypes. Overall, net photosynthesis rate, stomatal conductance, photosystem II efficiency and chlorophyll and starch content in leaves of the transgenic lines were indistinguishable from the WT counterparts (Table S12), which is in accordance with the fruit‐specific genetic manipulation.

As the transient starch accumulation during early tomato fruit development also directly influences total soluble sugar content at ripe stage (Davies and Cocking, 1965; Yin *et al.*, 2010), we next characterized the dynamics of starch accumulation in green WT and transgenic fruits. Both *PHYB2* and *PHYB2^Y252H^* overexpression negatively impacted starch accumulation, reducing the levels of this carbohydrate at IMG stage in 27% and 44%, respectively (Figure 5d, Table S11). The lower starch content in the transgenic lines at IMG stage was associated with a significant reduction in mRNA levels of the gene responsible for encoding the subunit L1 of ADP‐GLUCOSE PYROPHOSPHORYLASE (*AGPaseL1*) (Figure 5e), which is the AGPase large subunit‐encoding gene most expressed in tomato sink tissues (Bianchetti *et al.*, 2018; Petreikov *et al.*, 2010).

Although reduced starch synthesis in immature tomato fruits can also be caused by reduced sink capacity by the fruit tissues (Osorio *et al.*, 2014), the transcript abundance of key genes associated with tomato fruit sugar import and breakdown, such as *LYCOPERSICUM INVERTASE 5* and *6* (*LIN5* and *LIN6*) and *ACID INVERTASE 1* (*TIV1*), was not consistently altered in the transgenic lines compared with the WT during early fruit development (Table S13).

Taken together, our data suggest that sugar and lipid metabolisms are the major fruit primary metabolic pathways affected by fruit‐specific *PHYB2* or *PHYB2^Y252H^* overexpression. Rather than associated with changes in plant photosynthetic or fruit sink capacities, the reduced levels of soluble sugars in red ripe transgenic fruits seem to be associated with lower starch synthesis and accumulation during early fruit development.

### *PHYB2^Y252H^* overexpression leads to phytonutrient overaccumulation in fruits of distinct tomato genetic backgrounds

To evaluate whether fruit‐specific *PHYB2^Y252H^* overexpression can promote fruit quality regardless of the tomato genetic background, this phenomenon was also investigated in the well‐characterized tomato cultivar Ailsa Craig (AC). Wild‐type AC plants were crossed with Micro‐Tom (MT) wild‐type or homozygous *PHYB2‐* or *PHYB2^Y252H^*‐overexpressing plants generating F_1_ plants (AC‐WT, AC‐*PPC::B2* and AC‐*PPC::B2^Y252H^*, respectively) that were cultivated under greenhouse, semi‐controlled conditions until fruit harvesting.

**Figure 6:**
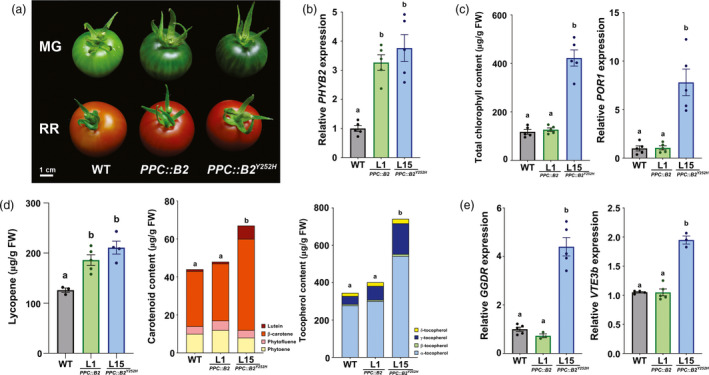
*PHYB2^Y252H^* overexpression also promotes fruit nutritional content in commercial tomato cultivar. (a) Visual phenotype of mature green (MG) and red ripe (RR) fruits of wild‐type (WT), *PPC2::PHYB2* (*PPC::B2*) and *PPC2::PHYB2^Y252H^* (*PPC::B2^Y252H^*) plants of Ailsa Craig cultivar growing under greenhouse conditions. (b) *PHYB2* or *PHYB2^Y252H^* mRNA levels in MG fruits. (c) Total chlorophyll content and *POR1* mRNA levels MG fruits. (d) Carotenoid (lycopene, phytoene, phytofluene, lutein and β‐carotene) and tocopherol (α, β, δ and γ forms) content in RR fruits. (d) *GGDR* and *VTE3b* mRNA levels in RR fruits. Transcript abundance was normalized against the WT samples. Data are mean ± SE, and dots represent individual values. Statistical differences are given by different letters (Tukey's test, α = 0.05). POR1, protochlorophyllide oxidoreductase; GGDR, geranylgeranyl diphosphate reductase; VTE3b, 2,3‐dimethyl‐5‐phytylquinol methyltransferase.

As observed for the MT background, MG fruits of AC plants overexpressing *PHYB2^Y252H^* exhibited darker green fruit coloration than AC‐WT, a phenotype explained by the higher chlorophyll accumulation and increased *POR1* transcript levels of the transgenic fruits (Figure 6a–c and S10). At the ripe stage, fruits from both AC‐*PPC::B2* and AC‐*PPC::B2^Y252H^* genotypes displayed higher lycopene accumulation and more intense red coloration than the AC‐WT counterparts (Figure 6a,d). Compared with AC‐WT, ripe AC‐*PPC::B2^Y252H^* fruits overaccumulated β‐carotene, lutein and all four (α, β, δ and γ) tocopherol forms (Figure 6d and S10). *PHYB2^Y252H^* overexpression in AC cultivar also resulted in the up‐regulation of key tocopherol biosynthetic genes as *GGDR* and *VTE3b* (Figure 6e). The levels of flavonoids, such as rutin and quercetin derivatives, and ascorbate were also increased in response to *PHYB2^Y252H^* overexpression, and reductions of around 10% in ºBrix were reported in red fruits of both AC‐*PPC::B2* and AC‐*PPC::B2^Y252H^* genotypes compared with the AC‐WT counterparts (Figure S10). Comparing data obtained from transgenic lines of MT and AC cultivars reveals that *PHYB2^Y252H^* overexpression can promote fruit nutritional quality in different tomato genetic backgrounds as well as distinct growth conditions (i.e. laboratory and greenhouse conditions).

## Discussion

Manipulation of light signalling pathway components has been increasingly investigated as a means to improve desired quality traits in many crops, including adjustments in plant architecture, shade avoidance responses, abiotic stresses tolerance and flowering time (Ganesan *et al.*, 2017; Gururani *et al.*, 2015). In tomato, accumulating genetic evidence indicates that alterations in downstream components of photoreceptor signal transduction pathways can impact fruit chemical composition by simultaneously affecting multiple metabolic pathways (Azari *et al.*, 2010b; Davuluri *et al.*, 2005; Liu *et al.*, 2004; Llorente *et al.*, 2016; Wang *et al.*, 2008). In contrast, attempts to promote tomato biofortification via increments in photoreceptor protein abundance (Giliberto *et al.*, 2005) or by genetic engineering photoreceptor properties (e.g. light sensitivity, protein stability) are currently scarce or missing, respectively. Here, we demonstrated that the fruit‐specific overexpression of the constitutively active Tyr^252^‐to‐His PHYB2 mutant version leads to the overaccumulation of multiple health‐promoting antioxidants in tomato fruits, regardless of the genetic background (i.e. Micro‐Tom or Ailsa Craig cultivars), without causing adverse collateral effects on vegetative development either when plants were grown under laboratory (i.e. Micro‐Tom) or greenhouse (i.e. Ailsa Craig) conditions. In contrast, overexpression of native *PHYB2* under the control of the same fruit‐specific promoter (*pPPC2*) resulted in less prominent, and sometimes non‐significative, increases in bioactive compounds (e.g. carotenoids, flavonoids, vitamin C and vitamin E) in ripe tomato fruits. These findings confirm that engineered PHY forms, particularly light‐independent constitutively active versions (Jeong *et al.*, 2016; Oka *et al.*, 2011; Su and Lagarias, 2007), represent a powerful photobiotechnological tool to increment the nutritional quality of fruits.

### Making room for more: PHYB2‐positive impacts on fruit plastid abundance and ultrastructure

The most striking visual phenotype of *PPC::B2* and particularly *PPC::B2^Y252H^* transgenic lines was the production of greener immature fruits and the persistence of the green shoulder during initial ripening, which were attributed to a higher abundance of chloroplasts in the pedicular region compared with the WT (Figure 1). In agreement, PHY‐mediated light perception has long been associated with chloroplast development and thylakoid formation from early seedling de‐etiolation throughout adult plant life (Girnth *et al.*, 1978; Mohr, 1977). In tomato fruits, PHY deficiency leads to reduced plastid size and density per cell (Bianchetti *et al.*, 2017), whereas the opposite phenotype has been observed in the light‐hyperresponsive tomato *high‐pigment* (*hp*) mutants *hp1* and *hp2* (Davuluri *et al.*, 2005; Kendrick *et al.*, 1997; Wang *et al.*, 2008). Fruit‐localized PHY deficiency has also been demonstrated to regulate mRNA levels of multiple tomato genes encoding key components of the plastid division machinery (Bianchetti *et al.*, 2018). Among these genes, *PDV2* was particularly up‐regulated in the *PHYB2*‐ or *PHYB2^Y252H^*‐overexpressing lines (Figure 2), which corroborates the proposed central role of this gene in determining plastid division rates in immature tomato fruits (Bianchetti *et al.*, 2018) and *Arabidopsis* leaves (Okazaki *et al.*, 2009).

In line with the identification of CURT1a protein accumulation as requisite for grana stacking in *Arabidopsis* leaf chloroplasts (Armbruster *et al.*, 2013; Pribil *et al.*, 2018), the up‐regulation of *CURT1a1* and *CURT1a2* in *PPC::B2^Y252H^* IMG fruits may be linked to the conspicuously incremented grana size registered in these transgenic lines (Figure 2). Also, genes associated with photosynthesis and chloroplast structure and functioning predominate among those up‐regulated in response to *PHYB2* or *PHYB2^Y252H^* overexpression (Figure 1), which agrees with the well‐reported positive influence of PHY signalling pathway on these gene categories in de‐etiolating seedlings and developing leaves (Dubreuil *et al.*, 2017; Hu *et al.*, 2009; Oh and Montgomery, 2014; Yoo *et al.*, 2019).

Increments in plastid ultrastructure positively affected isoprenoid accumulation once many active biosynthetic enzymes and their hydrophobic products are physically associated with plastid membranes (Llorente *et al.*, 2017; Yuan *et al.*, 2015). In tomato, such positive correlation is evident in several mutants and transgenic lines that produce plastid‐rich, dark green immature fruits, such as *hp1* and *hp2* mutants (Kolotilin *et al.*, 2007) and *GLK2*‐overexpressing lines (Lupi *et al.*, 2019; Powell *et al.*, 2012). Therefore, the increments in plastid abundance and ultrastructure observed in *PPC::B2* and *PPC::B2^Y252H^* fruits can be interpreted as a physical facilitator of isoprenoid biosynthesis and accumulation in the pericarp cells. However, many isoprenoid biosynthetic genes are also transcriptionally regulated by components of PHY signalling pathway (Gramegna *et al.*, 2018; Inagaki *et al.*, 2015; Llorente *et al.*, 2016), which possibly explains the differential impacts of *PHYB2* and *PHYB2^Y252H^* overexpression on the abundance of isoprenoids in green (chlorophylls) and ripening fruits (carotenoids and tocopherols). In fact, only *PPC::B2^Y252H^* fruits presented higher chlorophyll content than the WT, a differential response associated with the exclusive up‐regulation of chlorophyll biosynthetic genes in the *PPC::B2^Y252H^* lines (Figures 2, 3 and 6).

### PHYB2^Y252H^‐induced overaccumulation of bioactive compounds mainly relies on the up‐regulation of target biosynthetic genes

Tomato fruits are a major source of essential antioxidants in the human diet, such as carotenoids, tocopherols, flavonoids and ascorbate, which are associated with reducing the risks of cancer and cardiovascular diseases (Dorais *et al.*, 2008; Frusciante *et al.*, 2007). Many of these antioxidant classes accumulate or display altered composition as tomato fruit ripens via complex metabolic pathways tightly coordinated at the transcriptional level (Bovy *et al.*, 2007; Li *et al.*, 2019; Quadrana *et al.*, 2013; Verhoeyen *et al.*, 2002; Yuan *et al.*, 2015).

Carotenoids and tocopherols have as a precursor the MEP pathway‐derived GGDP (Pulido *et al.*, 2012). Transcriptional analysis revealed that *DXS1* and *GGPS2* were particularly up‐regulated in ripening *PPC::B2* and *PPC::B2^Y252H^* fruits, possibly boosting the production of GGDP to sustain higher carotenoid and tocopherol biosynthesis fluxes (Figure 3). Although *PHYB2^Y252H^* overexpression resulted in significantly higher levels of lycopene, β‐carotene and lutein, the most significant bioactive carotenoids in ripe tomatoes (Raiola *et al.*, 2014), core carotenoid biosynthetic genes (*e.g.* PSY1, PDS) were not consistently modulated at the transcriptional level in these transgenic lines (Figure 3). This seemingly contradictory result suggests that the *PHYB2^Y252H^*‐triggered up‐regulation of GGDP‐biosynthetic genes and/or the increased plastid abundance with more developed grana may suffice to sustain the higher carotenoid synthesis and accumulation detected in the ripe transgenic fruits. Alternatively, the presence of the constitutively active PHYB2^Y252H^ molecules may also have affected fruit carotenogenesis via additional regulatory levels.

Carotenoid‐related enzymes can also be post‐transcriptionally regulated, primarily relying on PQs as electron acceptors during the desaturation steps of phytoene and subsequent metabolites, driving lycopene biosynthesis (Norris *et al.*, 1995). *SPS*, a PQ biosynthetic gene, was up‐regulated in *PPC::B2^Y252H^* fruits, suggesting that PQ pool was increased in the transgenic fruits. Accordingly, silencing of tomato *SPS* reduces the levels of PQs, modifying carotenoid composition and leading to phytoene accumulation, possibly due to the impairment of desaturases activity (Jones *et al.*, 2013). The positive relation between the levels of PQs and carotenoid biosynthesis has also been reported in *Arabidopsis* (Kim *et al.*, 2015) and tomato *hp2* leaves (Jones *et al.*, 2013). The reduction of PQs is catalysed by the Ndh complex, essential for the control of carotenoid accumulation (Endo *et al.*, 2008). In tomato fruits, mutations in the *ORR* gene, encoding a Ndh subunit, led to decreased carotenoid content (Nashilevitz *et al.*, 2010), highlighting the importance of proper functioning of this redox chain to sustain tomato carotenogenesis. In line with this and in agreement with the enhanced carotenoids accumulation, *ORR* was up‐regulated in *PPC::B2^Y252H^* fruits.

The marked increase in total tocopherol content in the *PPC::B2^Y252H^* lines can be attributed to the up‐regulation of genes related to bottlenecks of PDP biosynthesis. *VTE5*, which catalyses the first and limiting step of channelling phytol chain into tocopherol biosynthetic pathway (Almeida *et al.*, 2016), was transcriptionally up‐regulated in *PPC::B2^Y252H^* BK fruits, providing PDP from chlorophyll turnover. Also, *GGDR* levels were up‐regulated in *PPC::B2^Y252H^* BK and RR fruits, possibly increasing PDP supply via MEP pathway. In line with our findings, the up‐regulation of *GGDR* in MG stages of *hp1* tomato mutants was also associated with increased tocopherol content in this mutant (Enfissi *et al.*, 2010). It has also been shown that *GGDR* transcription is negatively regulated by PIF3 in tomato fruits (Gramegna *et al.*, 2018). Thus, the intensification of PHYB2^Y252H^‐PIF3 interaction due to the constant presence of PHYB2^Y252H^ in pericarp cell nuclei, and consequent reduction in PIF3 protein accumulation, explains the markedly higher *GGDR* expression in *PPC::B2^Y252H^* from early stages of fruit development until the completion of ripening. Besides *GGDR*, *PHYB2^Y252H^* overexpression promoted the expression of *VTE1*, *VTE4* and *VTE3b*, the latter two already described to be positively correlated with fruit vitamin E content in tomato genotypes (Fritsche *et al.*, 2017; Quadrana *et al.*, 2013).

Light signals have been widely recognized as major environmental factors controlling flavonoid and vitamin C accumulation in tomato (Løvdal *et al.*, 2010; Massot *et al.*, 2012). In agreement, our findings indicate that the higher content of naringenin chalcone, rutin and kaempferol registered in the *PPC::B2^Y252H^* lines can be partially explained by the marked up‐regulation of *FLS* mRNA levels detected in the transgenic fruits at BK stage, when this gene is at peak expression (Figure 4c). Similarly, among all core ascorbate biosynthetic genes, only *GME1* was positively correlated with the vitamin C overaccumulation of *PPC::B2^Y252H^* fruits, corroborating the proposition of *GME1* expression as a major determinant of ascorbate levels in tomato fruits (Gilbert *et al.*, 2009; Li *et al.*, 2019; Massot *et al.*, 2012).

### Fruit primary metabolism is marginally affected by PHYB2 manipulation

Besides playing vital roles in primary metabolism, soluble sugars, organic acids and some aminoacids are also essential determinants of tomato fruit flavour (Carli *et al.*, 2009). Different from the overall PHYB2^Y252H^‐mediated changes in secondary metabolism, more limited differences in the abundance of primary metabolites were observed between the WT and transgenic ripe fruits, except for the over‐ and under‐accumulation of some lipids and soluble sugars, respectively (Figure 5). Such reduction in soluble sugars agrees with the slightly lower ^o^Brix values detected in ripe transgenic fruits, and the use of alternative promoters or further engineering in PHY sequence may be necessary to circumvent this negative collateral effect.

Accumulation of sugars in ripe tomato is directly influenced by the pool of starch synthesized at the early stages of fruit development, which is under the influence of multiple endogenous and environmental factors (Sagar *et al.*, 2013; Schaffer *et al.*, 2000; Yin *et al.*, 2010). After confirming that the fruit‐specific transgene overexpression had no impact on leaf photosynthetic capacity nor in the expression of predominant invertase genes, we identified a selective under‐accumulation of transcripts encoding a specific subunit of AGPase, an enzyme responsible for catalysing the first and rate‐limiting step of starch accumulation (Petreikov *et al.*, 2006; Stark *et al.*, 1992). In tomato, AGPaseL1 is the predominant large AGPase subunit in immature tomato fruits (Bianchetti *et al.*, 2018; Petreikov *et al.*, 2006), whose gene transcript accumulation is affected by fruit‐localized PHYs (Bianchetti *et al.*, 2018). Interestingly, *PHYB2* and *PHYB2^Y252H^* overexpression significantly reduced *AGPaseL1* mRNA levels specifically during early fruit development, when starch biosynthetic rates are maximum, suggesting a possible restriction in AGPase‐dependent production of the starch precursor ADP‐glucose.

As carbohydrate and fatty acids biosynthesis compete for the same carbon precursors (Rawsthorne, 2002; Yu *et al.*, 2018), it seems plausible to suggest a possible link between the lower sugar and higher lipid abundance in *PPC::B2^Y252H^* ripe fruits compared with the WT (Figure 5). Additionally, it has been demonstrated that tocopherol deficiency affects fatty acid metabolism, with consequences in sugar metabolism in tomato. By two distinct genetic interventions, tocopherol‐deficient plants showed higher amounts of starch and enhanced unsaturated fatty acid accumulation than WT. The changes in lipid profiles correlated with the reduction in *FADs* expression (Almeida *et al.*, 2016; Bermúdez *et al.*, 2018). Interestingly, an inverse scenario was described by our results. *PPC::B2^Y252H^* fruits showed higher tocopherol content, reduced amount of starch and up‐regulation of *FADs*. How tocopherol influences sugar metabolism has not yet been precisely addressed but it might be the result of the interaction between lipid and sugar metabolism machineries as observed here.

## Conclusion remarks

Our findings indicate that fruit‐specific overexpression of native *PHYB2* sequence is not sufficient to drive significant increments in health‐promoting secondary metabolites, a limitation resolved with the use of a light‐independent constitutively active version of this photoreceptor. The presence of continuously active PHYB2^Y252H^ molecules all over the fruit cells, regardless of the surrounding light conditions, probably leads to saturated PHYB2‐dependent light signalling. As a consequence, PHYB2 signalling‐dependent gene transcription is intensified, including many plastid‐related genes as well as biosynthetic enzymes responsible for the production of antioxidants. Experiments conducted using two genetic backgrounds (i.e. Micro‐Tom and Ailsa Craig) and two cultivation conditions (i.e. laboratory and greenhouse) allowed us to infer that *PHYB2^Y252H^* overexpression is a valid tool for promoting fruit nutritional content across tomato cultivars and at distinct growth conditions. Moreover, as phytochrome GAF domain Tyr residues are well conserved across plant species, the gain‐of‐function engineering of PHYs employed in this work may also be applicable as a means to promote biofortification in other fleshy fruits.

## Methods

### Plasmid constructs and tomato transformation

*Solanum lycopersicum PHYB2* full‐length coding sequence was amplified with Platinum SuperFi Green PCR Master Mix (Thermo Fischer Scientific, Waltham, USA) using tomato fruit cDNA as a template and the oligonucleotide sequences detailed in Table S14. The fragment was inserted into entry vectors pDON221 (Thermo Scientific) and recombined with pK7m24GW,3 and pEN‐L4‐PPC‐R1 plasmids (Fernandez *et al.*, 2009) via LR clonase II Plus (Thermo Scientific) reaction to generate the final construct *PPC2::PHYB2*. The single T‐to‐C base change required to modify the amino acid translation from Tyr^252^ to His^252^ was inserted using the QuikChange Lightning Site‐Directed Mutagenesis Kit (Agilent), resulting in the *PPC2::PHYB2^Y252H^* construct. The *Agrobacterium*‐mediated transformation of tomato (*Solanum lycopersicum* L.) plants cv. Micro‐Tom, which harbours the wild‐type *GLK2* allele (Carvalho *et al.*, 2011), was conducted as described by Bianchetti *et al. *(2018). Oligonucleotides used for plasmid construction and selection of transgenic plants are listed in Table S14. The homozygous Micro‐Tom (MT) lines were obtained from the T_2_ or T_3_ progeny by seeking 100% kanamycin resistance in the T_3_ or T_4_ seed population, respectively. Experiments were performed in homozygous T_4_ (or later) generations.

Homozygous MT *PHYB2* or *PHYB2^Y252H^*‐overexpressing lines were used to generate F_1_ plants in Ailsa Craig (AC) background. Flowers from wild‐type AC plants were emasculated at anthesis and hand‐pollinated with MT‐WT, MT‐*PPC2::PHYB2* or MT‐*PPC2::PHYB2^Y252H^* pollen. F_1_ seedlings were selected via kanamycin resistance assay, selected using oligonucleotides listed in Table S14.

### Growth conditions

Micro‐Tom wild‐type and transgenic lines were grown in a chamber under controlled conditions: 250 µmol m^−2^ s^−1^, 12‐h photoperiod, air temperature of 27°C day/22°C night and 60% day/80% night relative air humidity. Fruits were collected at immature green (IMG, on average 13 days post‐anthesis), mature green (MG, when green fruits are fully grown presenting locular gel), breaker (BK, when first signs of yellowing appear on the fruit bottom) and red ripe (RR, 12 days after BK) stages.

Ailsa Craig wild‐type and transgenic lines were grown under greenhouse, semi‐controlled conditions: average mean temperature of 25 ± 5°C, 11.5/13 light hours (winter/summer) and 250–350 mmol m^–2^ s^–1^ of incident photoirradiance. Fruits were harvested at MG and red ripe (RR, 12 days after BK) stages.

In all cases, fruits were harvested at the same time of the day (between the 4th and 6th hour of light period) in at least four biological replicates, with each replicate composed by at least four fruits from different plants. Seeds were removed, and the remaining tissues were immediately frozen in liquid nitrogen, powdered and stored at −80°C until use.

### RNA sequencing and data analysis

RNA extraction was performed as described in Bianchetti *et al. *(2018), and RNA quality was assessed using the Agilent 2100 Bioanalyzer (Agilent Technologies, Santa Clara, USA). Library preparation and sequencing using the Illumina HiSeq2500 system, as well as RNA‐seq assembly, annotation and differential expression analysis are detailed in Methods S1. Gene Set Enrichment Analysis (GSEA) was performed on Blast2GO software (version 5.2.5) (http://www.blast2go.org/) with the following modifications on default parameters: Gene Set Minimal Size and FDR filter value were set to 10 and 0.25, respectively.

### RT‐qPCR analysis

RNA extraction, cDNA synthesis and RT‐qPCR analyses were performed as described by Bianchetti *et al. *(2018). Oligonucleotide sequences used in the study are detailed in Table S14.

### Plastid ultrastructure and abundance

Plastid ultrastructure was assessed following methods described by Bianchetti *et al. *(2018). Pericarp sections from three immature fruits harvested from different plants were analysed per genotype. Plastid abundance was determined as described in Bianchetti *et al. *(2017). At least 15 individual cells were analysed per sample.

### Chlorophyll, carotenoid and tocopherol profiling

Chlorophyll *a*, chlorophyll *b*, phytoene, phytofluene, lycopene, β‐carotene and lutein levels were determined by high‐performance liquid chromatography with diode‐array detection (HPLC‐DAD) 1100 system (Agilent Technologies) as described in Cruz *et al. *(2018). Tocopherol extraction and quantification were performed as described in Lira *et al. *(2017). The endogenous metabolite concentration was obtained by comparing the peak areas of the chromatograms with commercial standards.

### Antioxidant activity, flavonoids and ascorbate quantification

For the antioxidant activity of polar extracts and flavonoid content analysis, approximately 100 mg fresh weight (FW) of powdered fruit pericarp samples was extracted with 1 mL of 80% (v:v) methanol for 30 min in an ultrasonic bath at room temperature followed by the collection of the supernatants by centrifugation (12 000 ***g***, 2 min, 25°C). DPPH (2,2‐Diphenyl‐1‐picrylhydrazyl) radical scavenging activity assay followed the protocol described by Furlan *et al. *(2015). Flavonoids were identified and quantified by HPLC systems according to the protocols and configurations described in Methods S1. Ascorbate extraction and detection were performed as described in Methods S1.

### Starch, soluble sugars and citrate quantification

Starch and soluble sugar extractions were performed as described by Bianchetti *et al. *(2017). Starch levels were determined from the dried pellet as described in Suguiyama *et al. *(2014). Citrate quantification followed the protocol described in Amóros *et al. *(2003).

### Leaf gas exchange and fluorescence parameters

Gas exchange parameters were measured between the 2nd and 4th hour of light period on the second fully expanded leaves from shoot to apex of approximately 2‐month‐old plants. Analyses were performed using a portable LI‐6400XTR infrared gas analyzer (LI‐COR Biosciences, Lincoln, USA) adjusted to a constant chamber temperature of 25°C, reference CO_2_ concentration of 400 ppm and photosynthetic photon flux density of 1000 µmol photons m^−2^ s^−1^. Chlorophyll fluorescence parameters were measured on the third fully expanded leaves from shoot to apex using a portable fluorometer MINI‐PAM (Heinz Walz GmbH, Effeltrich, Germany) following the protocol and derived calculations described in Alves *et al. *(2016).

### Metabolite profiling

Mass spectrometry (MS)‐based metabolite profiling followed the protocols described by Lisec *et al. *(2006) for polar compounds whereas that described in Bligh and Dyer (1959), Fiehn *et al. *(2000) and Ichihara and Fukubayashi (2010) was used for apolar compounds, with modifications described in Methods S1. Metabolite profile analyses were carried out in a gas chromatograph (Agilent Technologies 7890B) equipped with auto‐injector (CombiPAL, CG sampler 80) and a mass‐selective filter (Agilent Technologies 5977A). GC‐MS configurations are described in Methods S1.

### Statistical analyses

Experimental design was completely randomized. Statistical differences between groups were determined by ANOVA followed by Dunnett’s test for transcript abundance analyses (with WT as a control group, α = 0.05) or Tukey's HSD test (α = 0.05) for metabolites and remaining variables. Statistical analyses were performed using JMP statistical software package version 14 (https://www.jmp.com).

## Funding

This research was supported by the Fundação de Amparo à Pesquisa do Estado de São Paulo (FAPESP, #2016/04924‐0, #2016/01128‐9, #2018/25774‐2 and #2018/16389‐8), Conselho Nacional de Desenvolvimento Científico e Tecnológico (CNPq, #422287/2018‐0 and #305012/2018‐5) and Coordenação de Aperfeiçoamento de Pessoal de Nível Superior—Brazil (CAPES, Finance Code 001).

## Conflict of Interest

The authors declare no conflict of interest.

## Author contributions

FRRA, LF and MR designed the study; FRRA, BSL, FCP and SSM performed experiments; CMF, EP, GBP, SCSA and DD provided technical assistance and contributed to data analysis; FRRA and LF prepared the manuscript and collected contributions from all authors.

## Supporting information


**Figure S1 **Construct designed for the generation of transgenic lines.
**Figure S2 **Representative individual tomato plants from wild‐type and transgenic lines.
**Figure S3 **Validation of RNASeq analysis via RT‐qPCR.
**Figure S4 **TEM images of plastids of wild‐type and transgenic fruits.
**Figure S5 **Phylogenetic reconstruction of the CURT protein family.
**Figure S6 **Carotenoid and tocopherol profiles in wild‐type and transgenic fruits.
**Figure S7 **Flavonoid and ascorbate contents in wild‐type and transgenic fruits.
**Figure S8 **Simplified ascorbate biosynthetic pathway.
**Figure S9 **Brix and soluble sugar contents in wild‐type and transgenic fruits.
**Figure S10 **Impacts of *PHYB2* and *PHYB2^Y252H^*‐overexpression on Brix, isoprenoid, flavonoid, ascorbate composition in Ailsa Craig tomato cultivar.
**Method S1 **Extended Materials and Methods.

**Table S1** Transcript abundance of phytochrome‐encoding genes in wild‐type and transgenic fruits.

**Table S2** DEGs between transgenic and wild‐type fruits.

**Table S3 **Gene set enrichment analysis.

**Table S4 **Transcript abundance of plastid‐related genes in wild‐type and transgenic fruits.

**Table S5 **Chlorophyll, carotenoid and tocopherol content in wild‐type and transgenic fruits.

**Table S6 **Up‐regulated enzyme‐encoding DEGs involved in chlorophyll biosynthesis in tomato *PPC2::PHYB2^Y252H^* mature green (MG) fruits compared to wild‐type (WT).

**Table S7 **Transcript abundance of isoprenoid‐related genes in wild‐type and transgenic fruits.

**Table S8 **Antioxidant capacity and flavonoid contents in wild‐type and transgenic fruits.

**Table S9 **Transcript abundance of flavonoid and ascorbate‐related genes in wild‐type and transgenic fruits.

**Table S10 **Relative metabolite abundance registered in red ripe transgenic fruits.

**Table S11 **Starch, soluble sugar and citric acid contents in wild‐type and transgenic fruits.

**Table S12 **Photosynthetic parameters, chlorophyll and starch contents in leaves of wild‐type and transgenic plants.

**Table S13 **Transcript abundance of sugar‐related genes in wild‐type and transgenic fruits.

**Table S14 **Oligonucleotides used in this study.
