# Supplementary material for: Beyond the limits of photoperception: constitutively active PHYTOCHROME B2 overexpression as a means of improving fruit nutritional quality in tomato
Source: Plant Biotechnol J. 2020 Apr 1;18(10):2027–41. doi: 10.1111/pbi.13362 (PMC7540714; doi:10.1111/pbi.13362)
Supplement: Supplementary file 1 — Figure S1 Construct designed for the generation of transgenic lines. Figure S2 Representative individual tomato plants from wild‐type and transgenic lines. Figure S3 Validation of RNASeq analysis via RT‐qPCR. Figure S4 TEM images of plastids of wild‐type and transgenic fruits. Figure S5 Phylogenetic reconstruction of the CURT protein family. Figure S6 Carotenoid and tocopherol profiles in wild‐type and transgenic fruits. Figure S7 Flavonoid and ascorbate contents in wild‐type and transgenic fruits. Figure S8 Simplified ascorbate biosynthetic pathway. Figure S9 Brix and soluble sugar contents in wild‐type and transgenic fruits. Figure S10 Impacts of PHYB2 and PHYB2Y252H ‐overexpression on Brix, isoprenoid, flavonoid, ascorbate composition in Ailsa Craig tomato cultivar. Method S1 Extended Materials and Methods. [file PBI-18-2027-s014.docx]

**Beyond the limits of photoperception: constitutively active PHYTOCHROME B2 overexpression as a means of improving fruit nutritional quality in tomato**

**SUPPORTING INFORMATION**

**Figure S1.** Construct designed for the generation of transgenic lines.

**Figure S2.** Representative individual tomato plants from wild-type and transgenic lines.

**Figure S3.** Validation of RNASeq analysis via RT-qPCR.

**Figure S4.** TEM images of plastids of wild-type and transgenic fruits.

**Figure S5.** Phylogenetic reconstruction of the CURT protein family**.**

**Figure S6.** Carotenoid and tocopherol profiles in wild-type and transgenic fruits.

**Figure S7.** Flavonoid and ascorbate contents in wild-type and transgenic fruits.

**Figure S8.** Simplified ascorbate biosynthetic pathway.

**Figure S9.** °Brix and soluble sugars contents in wild-type and transgenic fruits.

**Figure S10.** Impacts of *PHYB2* and *PHYB2^Y252H^*-overexpression on °Brix, isoprenoid, flavonoid and ascorbate composition in Ailsa Craig tomato cultivar.

**Methods S1.** Extended Materials and Methods.

**Table S1.** Transcript abundance of phytochrome-encoding genes in wild-type and transgenic fruits. (Separate Excel file)

**Table S2.** DEGs between transgenic and wild-type fruits. (Separate Excel file)

**Table S3.** Gene set enrichment analysis. (Separate Excel file)

**Table S4.** Transcript abundance of plastid-related genes in wild-type and transgenic fruits. (Separate Excel file)

**Table S5.** Chlorophyll, carotenoid and tocopherol content in wild-type and transgenic fruits. (Separate Excel file)

**Table S6.** Up-regulated enzyme-encoding DEGs involved in chlorophyll biosynthesis in tomato *PPC2::PHYB2^Y252H^* mature green (MG) fruits compared to wild-type (WT). (Separate Excel file)

**Table S7.** Transcript abundance of isoprenoid-related genes in wild-type and transgenic fruits. (Separate Excel file)

**Table S8.** Antioxidant capacity and flavonoid contents in wild-type and transgenic fruits. (Separate Excel file)

**Table S9.** Transcript abundance of flavonoid and ascorbate-related genes in wild-type and transgenic fruits. (Separate Excel file)

**Table S10.** Relative metabolite abundance registered via non-targeted profiling in red ripe transgenic fruits. (Separate Excel file)

**Table S11.** Starch, soluble sugars and citric acid contents in wild-type and transgenic fruits. (Separate Excel file)

**Table S12.** Photosynthetic parameters, chlorophyll and starch contents in leaves of wild-type and transgenic plants. (Separate Excel file)

**Table S13.** Transcript abundance of sugar-related genes in wild-type and transgenic fruits. (Separate Excel file)

**Table S14.** Oligonucleotides used in this study. (Separate Excel file)


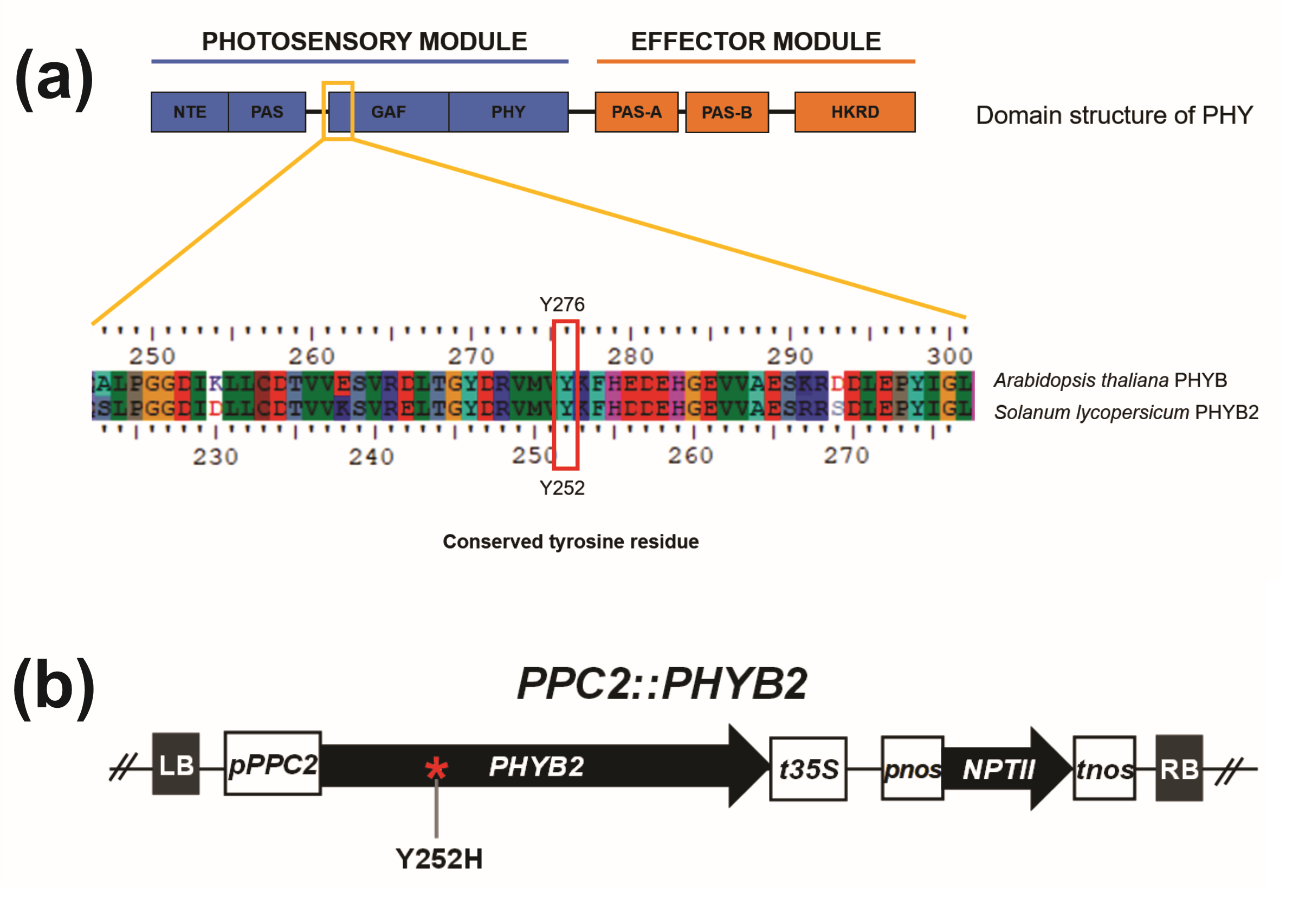


**Figure S1. Construct designed for the generation of transgenic lines.** (a) Detail of the alignment of *Arabidopsis* PHYB and tomato PHYB2. The conserved GAF domain Tyr residue is highlighted by the red box, which corresponds to Y^276^ and Y^252^ in *Arabidopsis* PHYB and tomato PHYB2, respectively. NTE, N-terminal extension domain; PAS, Per/Arnt/Sim domain; GAF, cGMP phosphodiesterase /Adenylcyclase/FhlA domain; PHY, phytochrome-specific domain; HKRD, histidine kinase-related domain. (b) Linearized representation of the vector used for the generation of transgenic *PPC2::PHYB2* (*PPC::B2*) and *PPC2:: PHYB2^Y252H^* (*PPC::B2^Y252H^*) lines. The tyrosine-to-histidine mutation is highlighted by the red asterisk. LB, left-border; pPPC2, *PHOSPHOENOL-PYRUVATE CARBOXYLASE 2* promoter; PHYB2, *PHYTOCHROME B2* coding sequence; t35S, 35S terminator; pnos, *NOPALINE SYNTHASE* gene promoter; responsible for the control of *NPTII* gene expression; *NPTII*, *NEOMYCIN PHOSPHOTRANSFERASE II* gene; tnos, nos terminator; RB, right-border.


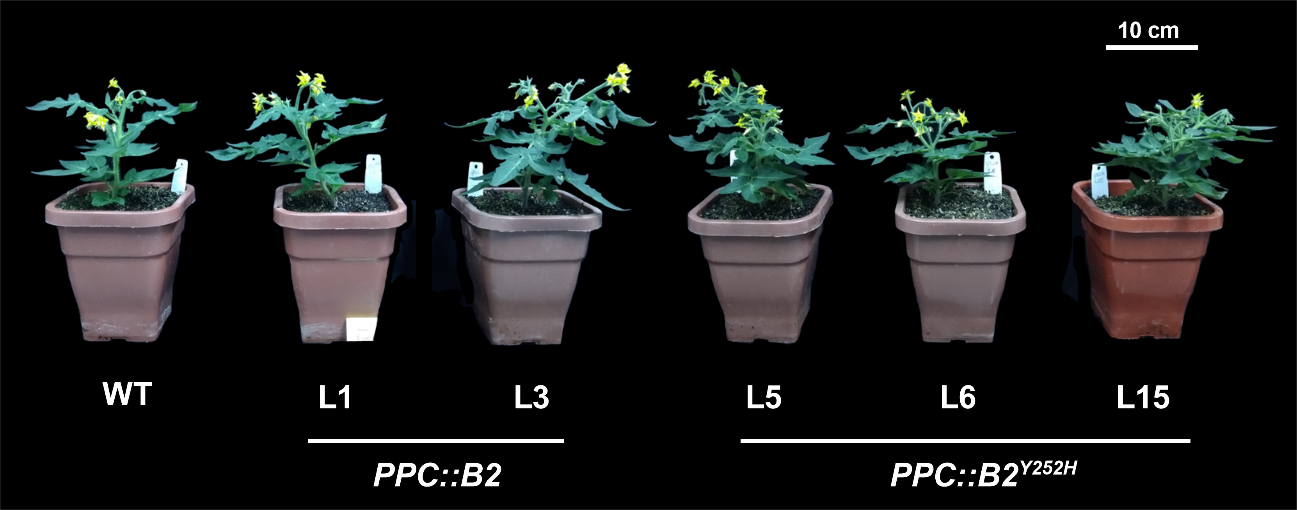


**Figure S2.** **Representative individual tomato plants from wild-type and transgenic lines.** Side view of one-month-old plants (early flowering stage) of Micro-Tom cultivar. WT, wildtype; *PPC::B2*, *PPC2::PHYB2*; *PPC::B2^Y252H^*, *PPC2::PHYB2^Y252H^*.


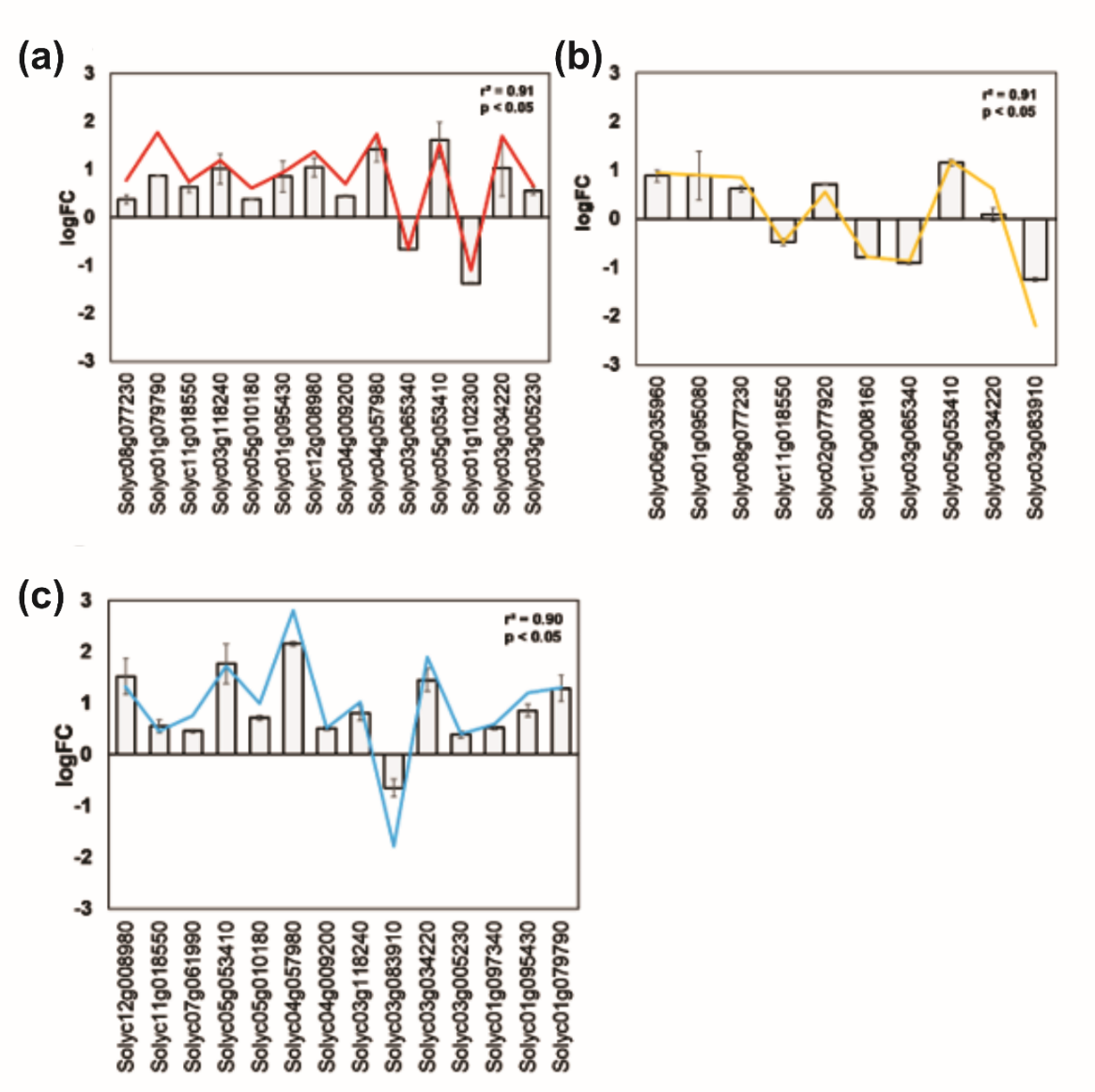


**Figure S3. Validation of RNASeq analysis via RT-qPCR.** Validation was performed for L6MGxWTMG (A), L1BKxWTBK (B) and L6BKxWTBK (C) comparisons as described in the Table S2. Bars represent mean log_2_ relative transcript abundances calculated in the RT-qPCR analyses. Lines represent mean log_2_ expression ratios calculated by pairwise DE analysis using edgeR. Pearson correlation was calculated for each comparison, and r^2^ and p-values are given in each figure.


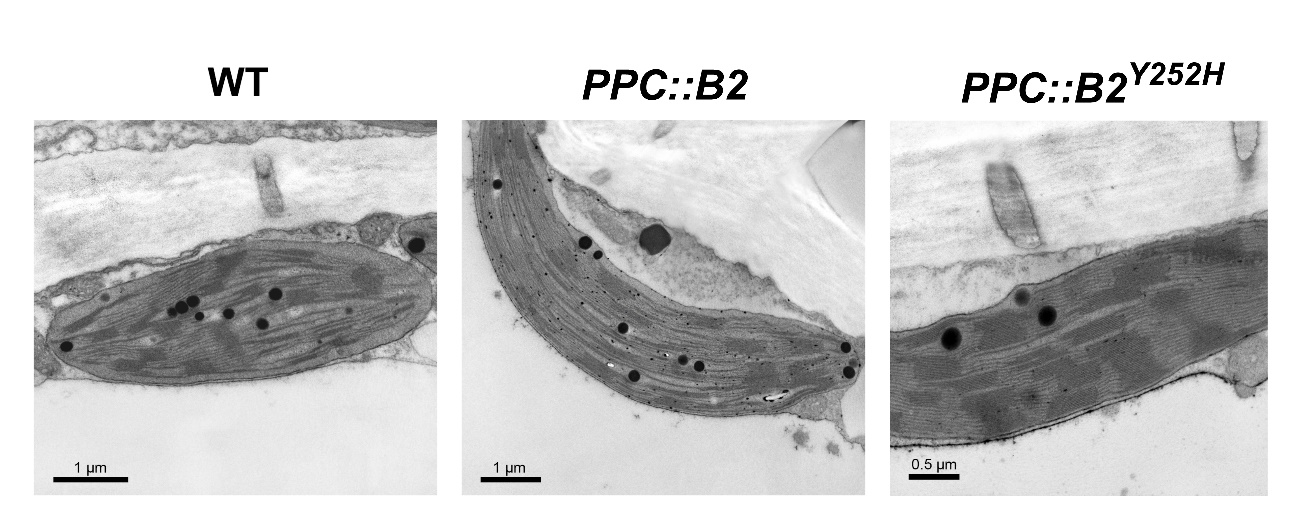


**Figure S4. TEM images of plastids of wild-type and transgenic fruits.** Transmission electronic microscopy (TEM) images of plastids of the pedicel region of immature fruits of wild-type (WT), *PPC2::PHYB2* (*PPC::B2*) and *PPC2::PHYB2^Y252H^* (*PPC::B2^Y252H^*) tomato plants of Micro-Tom cultivar.


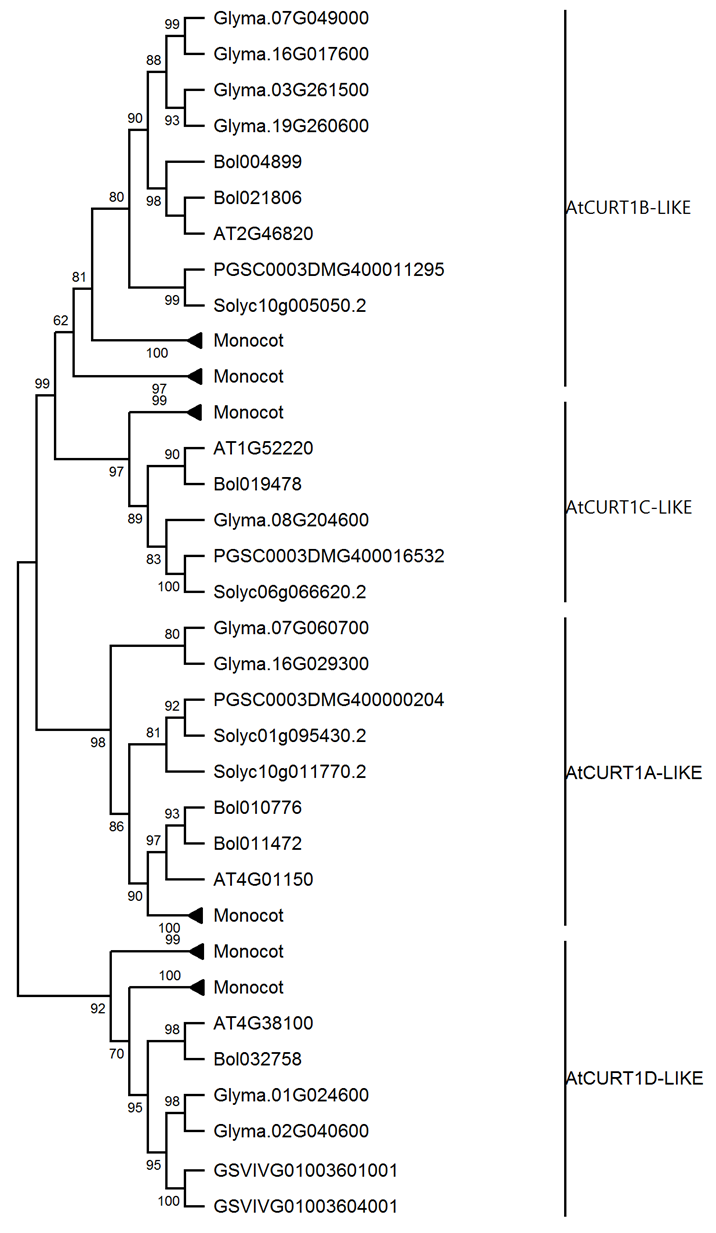


**Figure S5. Phylogenetic reconstruction of the CURT protein family.** Sequences from the families containing AtCURT proteins were retrieved from Phytozome 12.1 database (https://phytozome.jgi.doe.gov), aligned by ClustalW in MEGA 10.1 software^1^ and the tree was reconstructed with PhyML 3.0 algorithm^2^ with JTT substitution model and SH-like branch support. Monocot containing clades were compressed in the tree visualization. The groups were named accordingly to *Arabidopsis thaliana* sequences.

^1^ Kumar S *et al*. (2018) *Mol Biol Evol* **35**: 1547-1549.

^2^ Guindon S *et al*. (2010) *Syst Biol* **59**: 307-321.


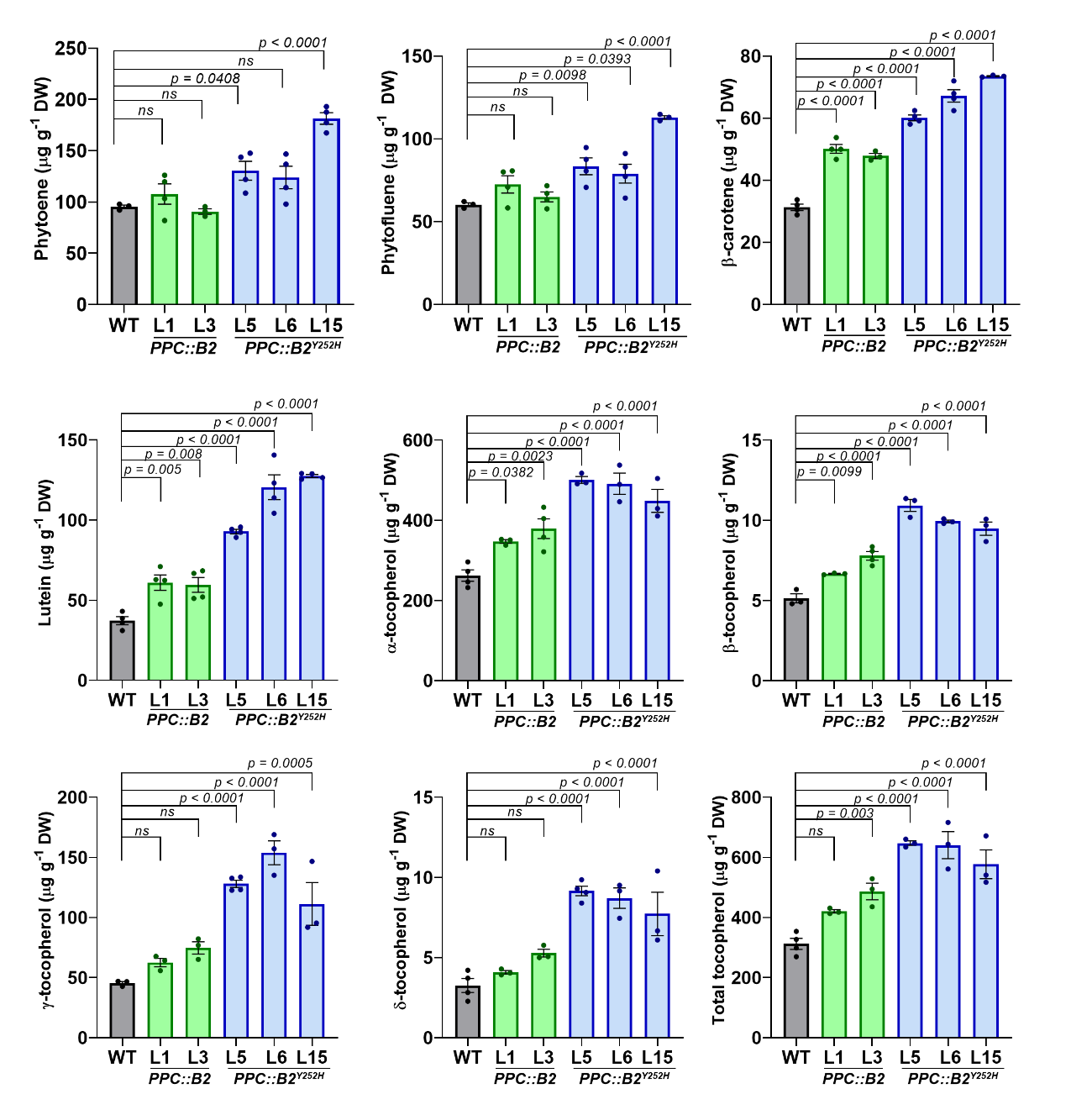


**Figure S6.** **Carotenoid and tocopherol profiles in wild-type and transgenic fruits.** Data obtained from red ripe fruits of wild-type (WT), *PPC2::PHYB2* (*PPC::B2*) and *PPC2::PHYB2^Y252H^* (*PPC::B2^Y252H^*) plants of Micro-Tom cultivar. Data are mean ± SE and dots represent individual values. *p* values are given for each comparison (Dunnett’s test with WT as control, α = 0.05). *ns*, non-significant; DW, dry weight.


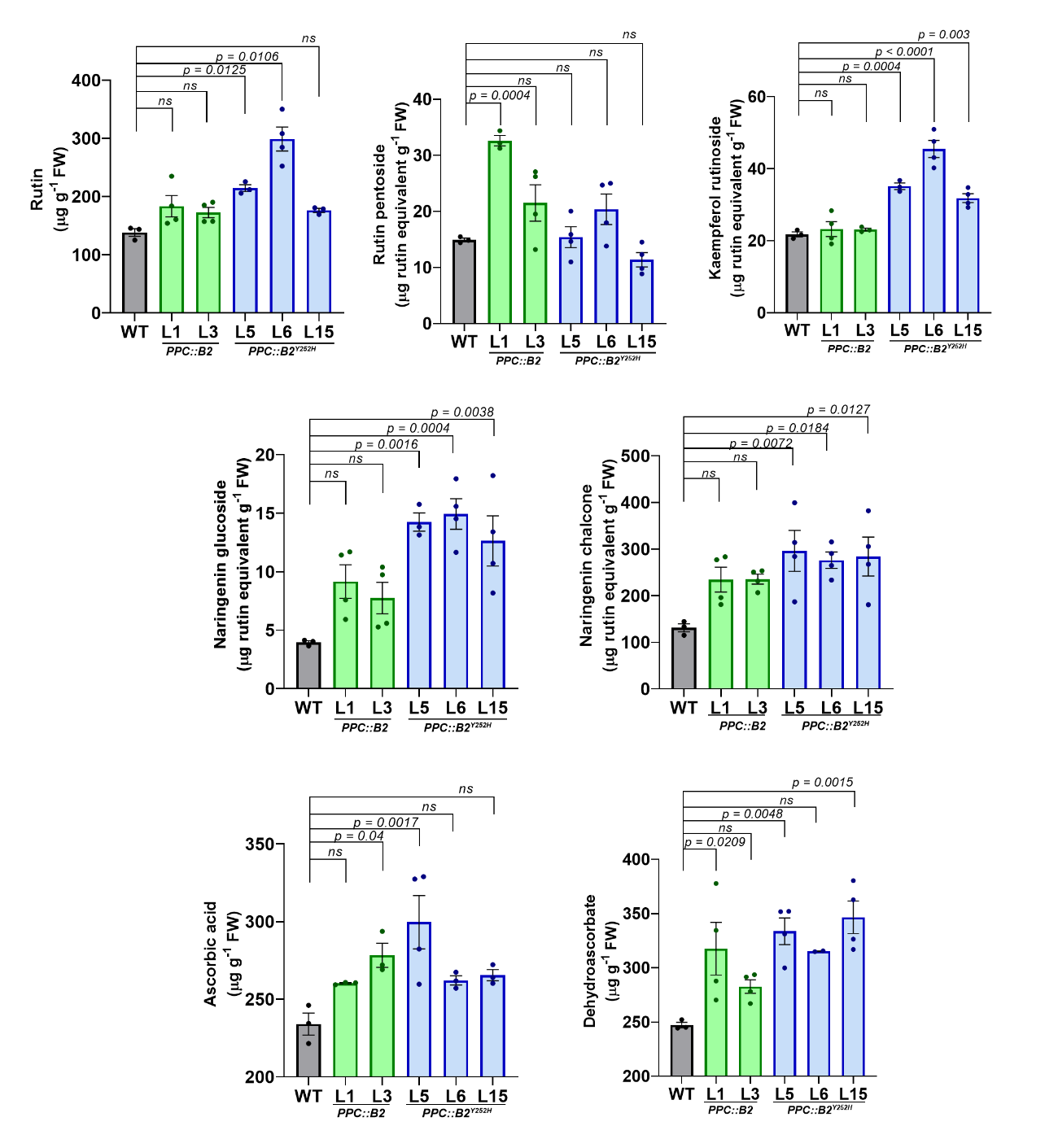


**Figure S7. Flavonoid and ascorbate contents in wild-type and transgenic fruits.** Data obtained from red ripe fruits of wild-type (WT), *PPC2::PHYB2* (*PPC::B2*) and *PPC2::PHYB2^Y252H^* (*PPC::B2^Y252H^*) plants of Micro-Tom cultivar. Data are mean ± SE, and dots represent individual values. *p* values are given for each comparison (Dunnett’s test with WT as control, α = 0.05). *ns*, non-significant; FW, fresh weight.


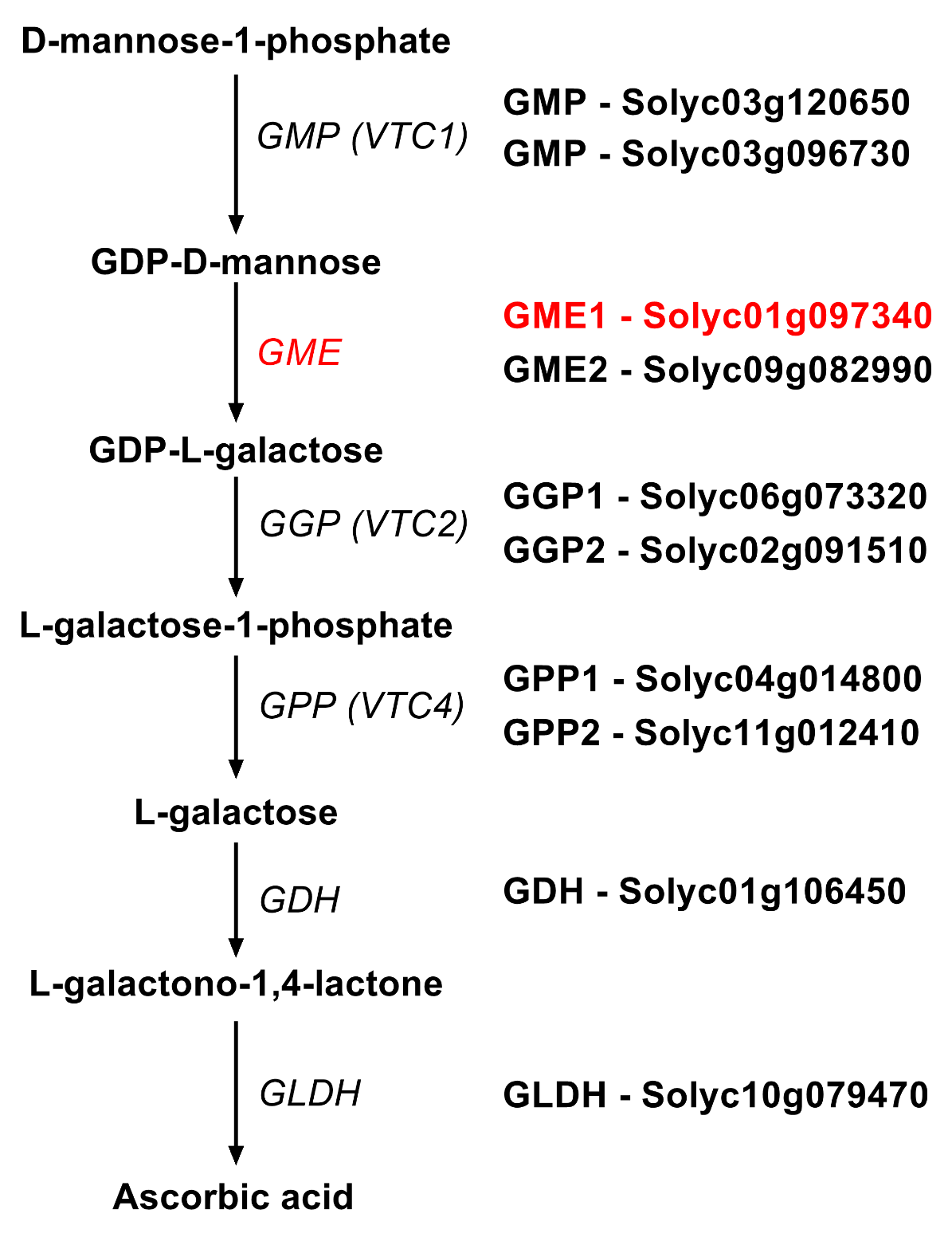


**Figure S8. Simplified ascorbate biosynthetic pathway.** Enzymes and corresponding gene IDs according to Sol Genomics Networks iTAG 3.2 annotation are given. Up-regulated enzyme-coding gene in *PPC::B2^Y252H^* fruits compared to the wildtype according to RNASeq analysis (Table S2) is highlighted in red. Gene abbreviations and relative transcript values are detailed in Table S9. GMP, GDP-D-mannose pyrophosphorylase; GME, GDP-D-mannose-3,5-epimerase; GGP, GDP-L-galactose pyrophosphorylase; GPP, L-galactose-1-phosphate phosphatase; GDH, L-galactose dehydrogenase; GLDH, L-galactono-1,4-lactone dehydrogenase.


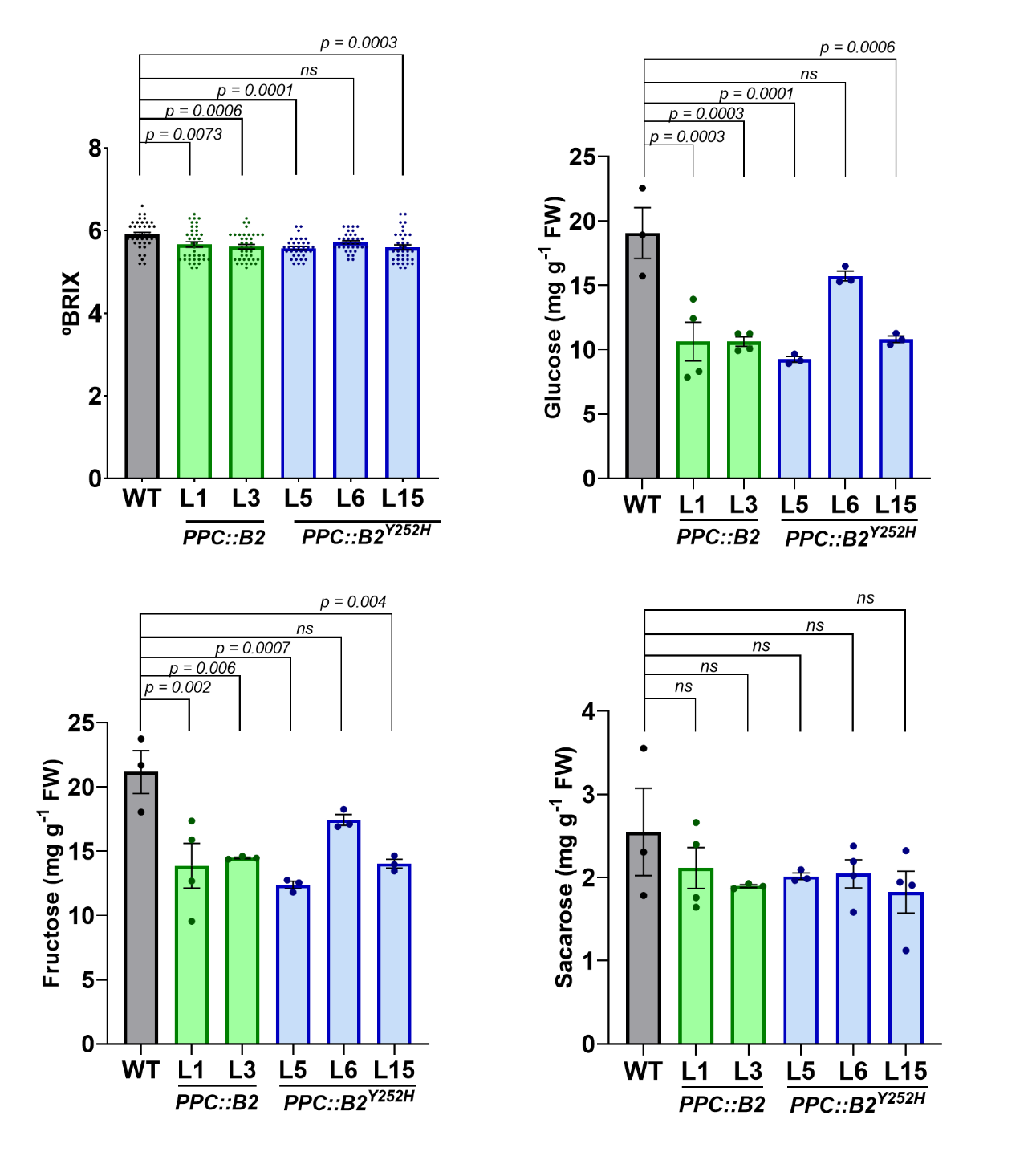


**Figure S9. °Brix and soluble sugars contents in wild-type and transgenic fruits.** Data obtained from red ripe fruits of wild-type (WT), *PPC2::PHYB2* (*PPC::B2*) and *PPC2::PHYB2^Y252H^* (*PPC::B2^Y252H^*) plants of Micro-Tom cultivar. Data are mean ± SE, and dots represent individual values. *p* values are given for each comparison (Dunnett’s test with WT as control, α = 0.05). *ns*, non-significant; FW, fresh weight.


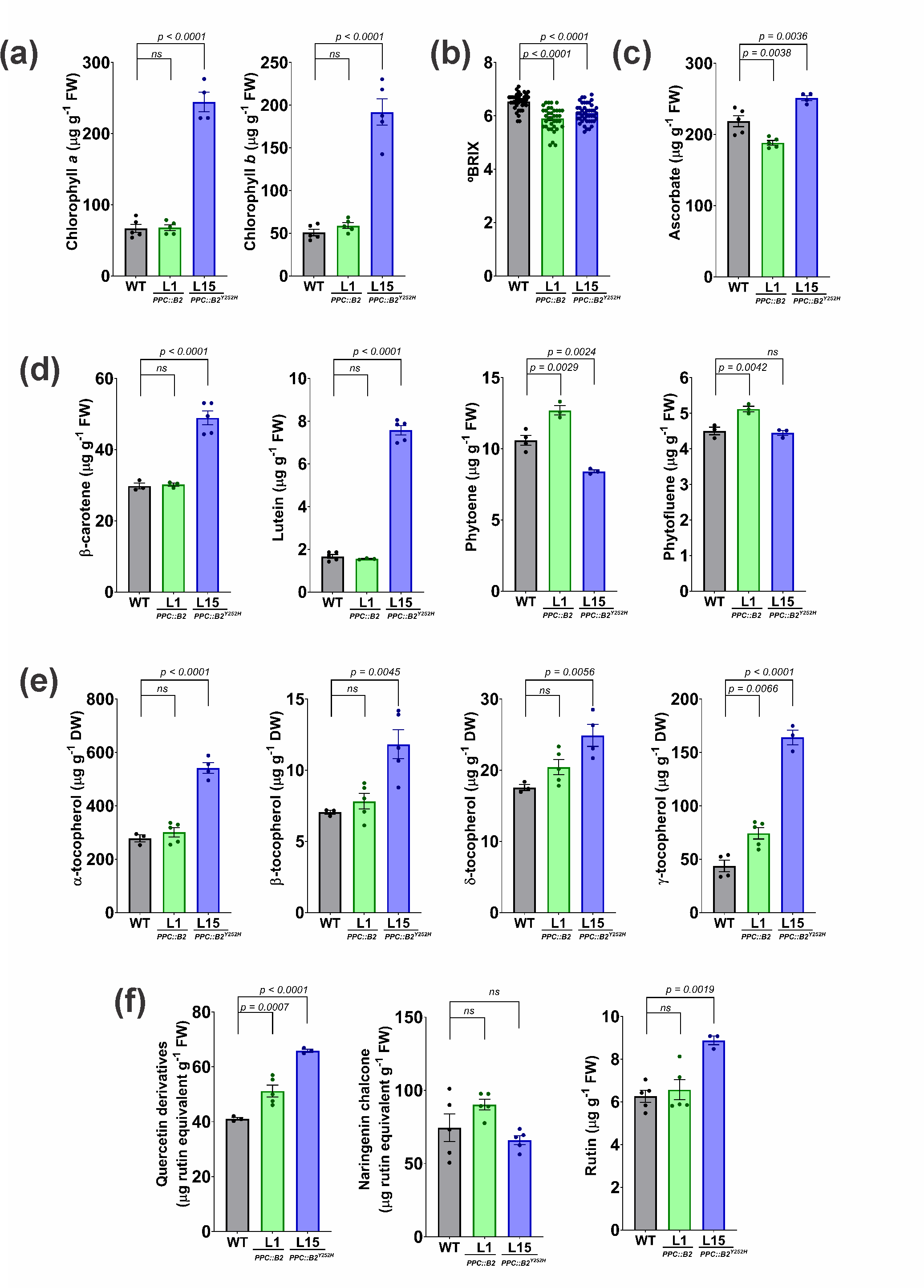


**Figure S10. Impacts of *PHYB2* and *PHYB2^Y252H^*-overexpression on °Brix, isoprenoid, flavonoid and ascorbate composition in Ailsa Craig tomato cultivar.** (a) Chlorophyll *a* and *b* content in fruits of wild-type (WT), *PPC2::PHYB2* (*PPC::B2*) and *PPC2::PHYB2^Y252H^* (*PPC::B2^Y252H^*) plants of Ailsa Craig tomato background. (b) °Brix. (c) Ascorbate content. (d) Carotenoid composition. (e) Tocopherol composition. (f) Flavonoid composition. Data are mean ± SE, and dots represent individual values. *p* values are given for each comparison (Dunnett’s test with WT as control, α = 0.05). *ns*, non-significant; FW, fresh weight; DW, Dry weight.

**Methods S1** Extended Materials and Methods.

***RNA sequencing, reads mapping and differential expression analysis***

RNA sequencing was performed using Illumina HiSeq 2500 System. The filtering of low-quality reads, primers sequences and vectors were performed by the program Seqyclean v.1.9.10 (https://bitbucket.org/izhbannikov/seqyclean), using as cutoff bases with average quality lower than 24QScore. The Univec database was used to filter contaminants out (<http://www.ncbi.nlm.nih.gov/VecScreen/> UniVec.html). After filtering, reads with length less than 65pb were removed. Filtered reads were mapped and counted using the package STAR 2.6.1 (Dobin *et al*., 2013) to the genome sequences of *Solanum lycopersicum* downloaded from SOL Genomics Network database (ITAG3.2 version, SGN, <http://solgenomics.net/organism/Solanum_lycopersicum/genome>).

Only the uniquely mapped reads were used for differential expression analysis. The edgeR program (Robinson *et al*., 2010) from R/Bioconductor package was used to obtain the differentially expressed genes. Within the program, the data from each group were normalized with TMM (Robinson and Oshlack, 2010) using the calcNormFactors function. Contigs with expression profiles of zero in at least three samples were removed in order to avoid artifacts caused by low expression contigs. Differential expression analysis was performed using the negative binomial function, applying the Benjamini-Hochberg correction (Benjamini and Hochberg, 1995) for multiple tests to avoid false positives. Only genes with FDR < 0.05 were considered differentially expressed.

***Flavonoid quantification and identification***

Flavonoid compounds were identified by HPLC-DAD 1260 system (Agilent Technologies, USA) equipped with an autosampler, using a Zorbax C18 column (150 x 4.6 mm, 3.5 µm particle diameter) at 45ºC with a flow rate of 1 mL min^-1^. The mobile phase was a gradient of 0.1% acetic acid (A) and acetonitrile (B): 0 to 6 min with 85% A:15% B, 6 to 20 min with 70% A: 30% B, 20 to 30 min with 100% B. Eluted compounds were detected and quantified at 280 and 352 nm wavelengths using rutin as a standard (1.5 to 150 µg mL^-1^). For compound identification, samples were analyzed by HPLC (CBM-20A Shimadzu) coupled to a mass spectrometer (Amazon Speed EDT, Bruker Daltonics) using the same column, solvent gradient and conditions cited above. Specific MS configuration was nebulizer at 27 Psi, dry gas of 12 L min^-1^, temperature of 325 ºC, and HV of 4500V. Data were acquired in the positive and negative ion modes. The full-scan mass acquisition was performed by scanning in the range m/z 100–900. The endogenous metabolite concentration was obtained by comparing the peak areas of the chromatograms with commercial standards.

***Ascorbate extraction and quantification***

For ascorbate extraction, approximately 200 mg FW of powdered fruit pericarp samples was vortexed with 2 mL of extraction buffer composed by 6% (v:v) HPO_3_ and 1mM EDTA for 1 min and the supernatant was collected after centrifugation (10000 rpm, 15 min, 4ºC). For the quantification of reduced ascorbate, sample extracts were diluted 1:20 in H_3_PO_4_ (pH 2.3). For the quantification of total ascorbate pool, 100 uL of sample extracts were vortexed with 20 µL of 0.2% (w/v) DTT and 10 µL of 45% (w/v) K_2_HPO_4_ for 1 min and maintained under dark conditions for 20 min. Then, 20 µL of 2M H_3_PO_4_ and 350 µL _d_H2O were added to the samples. Ascorbate was quantified on an HPLC-DAD 1260 system (Agilent Technologies, USA) equipped with an autosampler, using a Zorbax Eclipse XDB-C18 column (150 x 4.6 mm; 5 µm particle diameter) with a flow rate of 1 mL min^-1^. The mobile phase was H_3_PO_4_ (pH 2.3) isocratic for 4 min. Eluted compounds were detected and quantified at 245 and 194 nm wavelengths. Dehydroascorbate content was quantified by the difference between total and reduced ascorbate contents using ascorbate as a standard (0.8 to 200 µM). The endogenous metabolite concentration was obtained by comparing the peak areas of the chromatograms with commercial standards.

***Apolar metabolite profiling***

For apolar metabolite extraction, 1g FW of powdered fruit pericarp was mixed with 2.5 mL of chloroform: methanol solution at a proportion of 1:2 (v:v) added with 65 µg of tridecanoic acid as the internal standard. The mixture was extracted for 30 min at 4°C under agitation, mixed with 1.25 mL chloroform and 1.25 mL sodium sulfate 1.5% (m:v) and shaken for further 5 min at 4°C. After centrifugation at 1,000 *g* for 5 min at 4°C, the upper phase was discarded, 0.25 g of sodium sulfate was added, vortexed and centrifuged at 2,000 *g* for 5 min. The supernatant was transferred to appropriated vials and reduced to dryness under N_2_ flow. For the first derivatization, samples were resuspended in 1.2 mL of hexane:toluene 5:1 (v:v), 1.5 mL methanol and 300 µL HCl 8% (v:v methanol), vortexed for 30 s and incubated for 90 min at 100°C. Sequentially, 1 mL of hexane and 1 mL of _d_H_2_O were added to the extract, vortexed for 30 s and dried under N_2_ flow. For the second derivatization, samples were resuspended in 240 µL hexane, 20 µL pyridine and 20 µL MSTFA (N-methyl-N-trimethylsilyl-trifluoroacetamide), vortexed until complete solubilization and transferred to vials for GC-MS injection.

***Metabolite profiling GC-MS configuration***

Metabolites were separated in a HP 5MS UI column (30m x 0.25 mm x 0.25 µm/ Agilent Technologies, USA). Samples were injected at 250°C in splitless mode using helium as carrier gas under 1 mL min^-1^ flow rate. Column temperature programming set was adjusted at 60°C for 1 min, ramp temperature of 5°C per minute until 325°C, maintained for 10 min. MS parameters were: ion source set at 230°C, interface set at 290°C, mass range 50-600 m/z scanned at 2.7 scans per second. Mass spectra of the compounds were compared to the library of the National Institute of Standards and Technology (NIST/EPA/NIH, 2011) using the Agilent MassHunter Workstation software (version B.06.00, Agilent, CA, USA).

***References - Extended Materials and Methods***

Benjamini Y, Hochberg Y (1995) Controlling the false discovery rate: a practical and powerful approach to multiple testing. *Journal of the Royal Statistical Society* **57**: 289-300.

Dobin A, Davis CA, Schlesinger F, Drenkow J, Zaleski C, Jha S, Batut P, Chaisson M, Gingeras TR (2013) STAR: ultrafast universal RNA-seq aligner. *Bioinformatics* **29**: 15-21.

Robinson MD, McCarthy DJ, Smyth GK (2010) edgeR: a Bioconductor package for differential expression analysis of digital gene expression data. *Bioinformatics* **26**: 139-140.

Robinson MD, Oshlack A (2010) A scaling normalization method for differential expression analysis of RNA-seq data. *Genome Biology* **11**: R25.
